# Supplementary material for: Roles of three cytochrome P450 monooxygenases in triterpene biosynthesis and their potential impact on growth and development
Source: Plant Physiol. 2024 Jul 25;196(2):1407–25. doi: 10.1093/plphys/kiae399 (PMC11444297; doi:10.1093/plphys/kiae399)
Supplement: kiae399_Supplementary_Data [file kiae399_supplementary_data.zip › Supporting information of figures_edited_submit.docx]

## Supplementary Information

**Article title:** Roles of cytochrome P450 monooxygenases in triterpene biosynthesis and their potential impact on growth and development

Authors: Caiqiong Yang, Rayko Halitschke, Sarah E. O'Connor, and Ian T. Baldwin



**Supplementary Figure S1** **p450 enzymes from *N. attenuata* plants**

The *Nicotiana attenuata* genome was downloaded from the Ensembl Plants database (http://plants.ensembl.org/info/data/ftp/index.html). The Hidden Markov Model (HMM) of the conserved structural domain of cytochrome P450 (PF00067) was obtained from the Pfam database (https://www.ebi.ac.uk/interpro/). The microarray data originated from the *Nicotiana attenuata* data hub (Brockmöller et al., 2017). Experimental conditions and tissues are abbreviated as follows: ROT: root treated with *Manduca sexta* oral secretion, LET: leaf treated with *M. sexta* oral secretion, LEC: leaf control, SED: seed; STT: stem treated with *M. sexta* oral secretion, COE: corolla early, COL: corolla late, STI: stigma, POL: pollen tubes, SNP: style without pollination, ANT: Anthers, NEC: nectaries, OVA: ovary, PED: pedicels, OFL: opening flower, FLB flower buds. The blue-colored plus signs indicate the P450 candidates involved in triterpenoid biosynthesis. The amino acid sequences of cytochrome P450 genes were aligned using ClustalW with default parameters, as implemented in MEGA 11 software. A neighbor-joining tree was constructed and subjected to bootstrapping analysis (1,000 iterations). Distances were computed using the Jones-Taylor-Thornton matrix-based method. The scale bar indicates the branch length representing 0.9 amino acid substitutions per site. The nodes along the branches represent bootstrap values, denoting the statistical support for the tree's topology in a phylogenetic context.


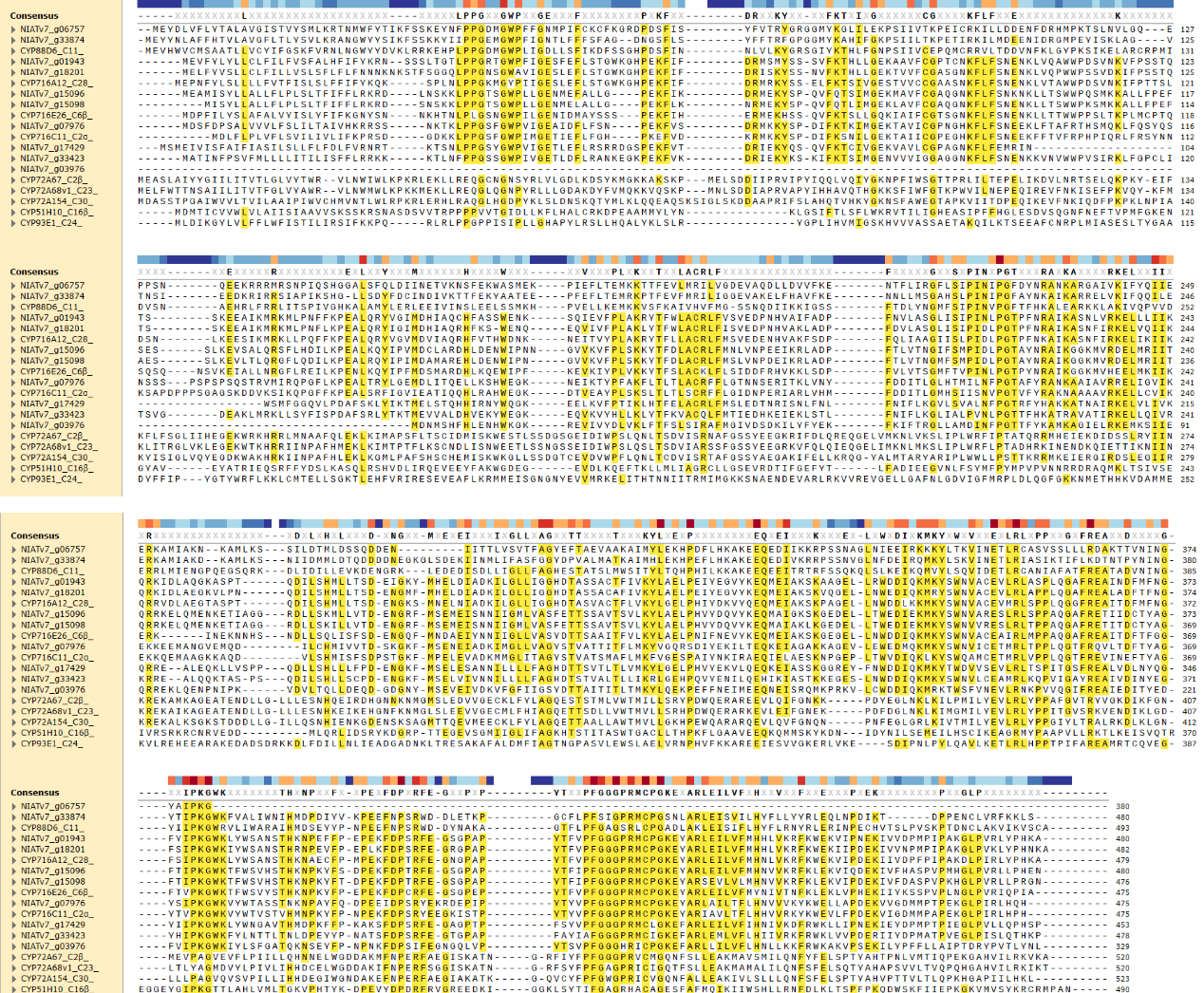


**Supplementary Figure S2** **Sequence alignment of *N. attenuata* CYP450 candidates with CYP450 enzymes from the triterpenoid biosynthetic pathway.** The amino acid sequences of CYP450 candidates derived from the *Nicotiana attenuata* Data Hub (http://nadh.ice.mpg.de/NaDH/), while the amino acid sequences of CYP450 enzymes in the triterpenoid biosynthetic pathway are sourced from the NCBI database. The sequence is annotated with colored bars indicating conserved amino acid residues. The heatmap at the top of the sequence illustrates the conservation level of residues, with warmer colors indicating higher conservation levels and cooler colors indicating lower levels.





**Supplementary Figure S3 The levels of triterpenes after co-expression of *AtLPU1/NaOSC2* with different CYP450s.** N1: β-amyrin, N2: erythrodiol, N3: putative 2α-hydroxy β-amyrin, N4: oleanolic acid, N5: oleanolic aldehyde, N6: daturadiol, A1: lupeol, A2: putative 2α-hydroxy lupeol, A3: betulin, A4: betulinic acid, A5: 3alpha,20-lupanediol, A6: putative 28-hydroxy lupanediol, A7: putative 2α-hydroxy lupanediol, A8: putative 28-acrboxy lupanediol, A9: putative 28-aldehyde lupanediol. The central line within the box represents the median of the data. The box's upper and lower boundaries denote the data's upper and lower quartiles. The lines above and below the box, known as whiskers, signify the variability of the data (error bar). The whiskers’ length is set at 1.5 times the interquartile range. The points represent specific observations in the data set. The values beyond 1.5 times the interquartile range are considered as outliers. ANOVAs with Tukey's test and independent Samples Kruskal-Wallis test were used for statistical analyses with a significance level of 0.05 (n=3, mean ± SE,different letters indicate significant differences between the two groups at the 0.05 level)

**

**

**Supplementary Figure S4 Primary fragmentation pathways of TMS-derivatives of oleanolic aldehyde.** The structure marked in red is the C28 oxidation site. -TMSOH: trimethylsilanol. -CHO: aldehyde group. RDA: retro-Diels–Alder cleavage.





**Supplementary Figure S5 Depicts the triterpenoid metabolite profiles of *N. benthamiana* leaves expressing either empty vector (EV) or *NaCYP716A419*. (A)** Triterpenoid metabolite profiles of *N. benthamiana* leaves co-expressing NaCYP716A419 with either EV or NaOSC2 (SIM: 189, 203, 320, 216). **(B)** Triterpenoid metabolite profiles of *N. benthamiana* leaves expressing EV after feeding with either 0.1% DMSO or 100μM erythrodiol (SIM: 189, 203, 216). In panel A, erythrodiol (N2), oleanolic acid (N4), and maslinic acid (N7) were used in authentic standard 1, and β-amyrin (N1) and lupeol (A1) were used in authentic standard 2. In panel B, β-amyrin (N1), erythrodiol (N2), and oleanolic acid (N4) were used as authentic standards.


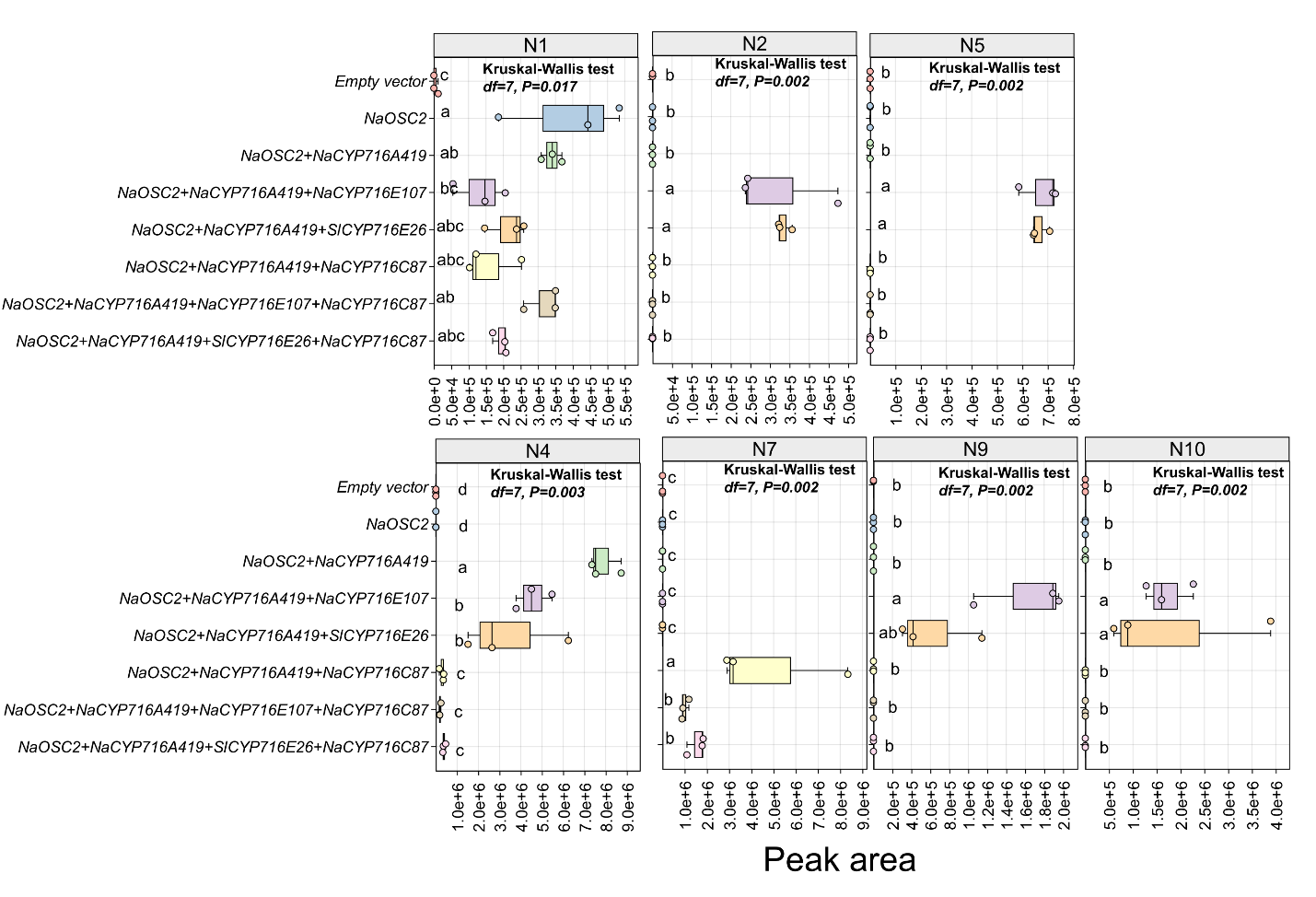


**Supplementary Figure S6 The levels of triterpenes after co-expression of *NaOSC2* and *NaCYP716A419* with *NaCYP716E107, SlCYP716E26,* or *NaCYP716C87*.** N1: β-amyrin, N2: erythrodiol, N4: oleanolic acid, N5: oleanolic aldehyde, N7: maslinic acid, N9: putative 6β-hydroxy oleanolic acid, N10: putative incomplete derived 6β-hydroxy oleanolic acid. The central line within the box represents the median of the data. The box's upper and lower boundaries denote the data's upper and lower quartiles. The lines above and below the box, known as whiskers, signify the variability of the data (error bar). The whiskers’ length is set at 1.5 times the interquartile range. The points represent specific observations in the data set. The values beyond 1.5 times the interquartile range are considered as outliers. ANOVAs with Tukey's test and independent Samples Kruskal-Wallis test were used for statistical analyses with a significance level of 0.05 (n=3, mean ± SE, different letters indicate significant differences between the two groups at the 0.05 level.)





**Supplementary Figure S7** **Primary fragmentation pathways of TMS-derivatives of β-amyrin.** The structure marked in red is the C28 oxidation site. -TMSOH: trimethylsilanol. -TMSCOOH: trimethylsilyl acetic acid. RDA: retro-Diels–Alder cleavage.





**Supplementary Figure** **S8 Primary fragmentation pathways of TMS-derivatives of maslinic acid.** The structure marked in red is the C28 oxidation site, the structure marked in blue is the C2α oxidation site. -TMSOH: trimethylsilanol. -TMSCOOH: trimethylsilyl acetic acid. -TMSCO: trimethylsilyloxy group. RDA: retro-Diels–Alder cleavage.





**Supplementary Figure S9 Primary fragmentation pathways of TMS-derivatives of erythrodiol.** The structure marked in red is the C28 oxidation site. -TMSOH: trimethylsilanol. RDA: retro-Diels–Alder cleavage.





**Supplementary Figure S10 Primary fragmentation pathways of TMS-derivatives of lupeol.** The structure marked in red is the C28 oxidation site. -TMSOH: trimethylsilanol. RDA: retro-Diels–Alder cleavage.





**Supplementary Figure S11 Primary fragmentation pathways of TMS-derivatives of lupanediol.** The structure marked in red is the C28 oxidation site. -TMSOH: Trimethylsilanol. -TMSCO: Trimethylsilyloxy group. RDA: retro-Diels–Alder cleavage.


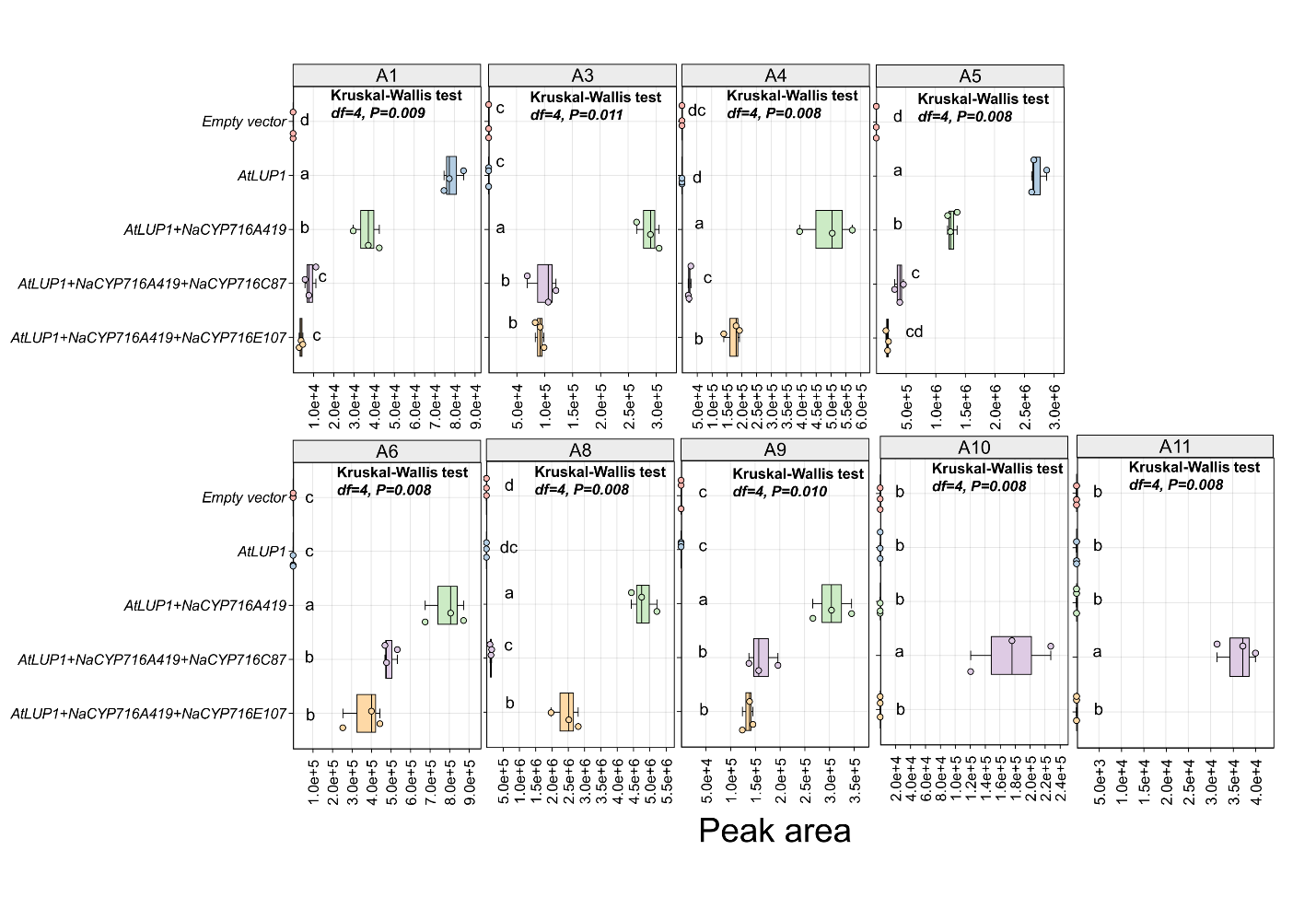


**Supplementary Figure S12 The levels of triterpenes after co-expression of *AtLPU1* and *NaCYP716A419* with *NaCYP716E107* or *NaCYP716C87*.** A1: lupeol, A3: betulin, A4: betulinic acid, A5: 3alpha,20-lupanediol, A6: putative 28-hydroxy lupanediol A8: putative 28-carboxy lupanediol, A9: putative 28-haldehyde lupanediol, A10: putative alphitolic acid, A11: putative 2α-hydroxy 28-carboxy lupanediol. The central line within the box represents the median of the data. The box's upper and lower boundaries denote the data's upper and lower quartiles. The lines above and below the box, known as whiskers, signify the variability of the data (error bar). The whiskers’ length is set at 1.5 times the interquartile range. The points represent specific observations in the data set. The values beyond 1.5 times the interquartile range are considered outliers. ANOVAs with Tukey's test and independent Samples Kruskal-Wallis test were used for statistical analyses with a significance level of 0.05 (n=3, mean ± SE, different letters indicate significant differences between the two groups at the 0.05 level.)





**Supplementary Figure** **S13 Primary fragmentation pathways of TMS-derivatives of oleanolic acid.** The structure marked in red is the C28 oxidation site. -TMSOH: trimethylsilanol. -TMSCOOH: trimethylsilyl acetic acid. -TMSCO: trimethylsilyloxy group. RDA: retro-Diels–Alder cleavage.





**Supplementary Figure S14 Primary fragmentation pathways of TMS-derivatives of betulinic acid.** The structure marked in red is the C28 oxidation site. -TMSOH: Trimethylsilanol. -TMSCOOH: Trimethylsilyl acetic acid. RDA: retro-Diels–Alder cleavage.





**Supplementary Figure S15 Alignment of protein sequences of NaCYP716E107, SlCYP716E26, and CaCYP716E41.** The sequence is annotated with different colored bars indicating amino acid residues with distinct characteristics. At the top of the sequence, a heatmap illustrates the conservation level of residues, with warmer colors indicating higher conservation levels and cooler colors indicating lower levels.





**Supplementary Figure S16 The levels of triterpenes after expressing *NaCYP716E107* or *SlCYP716E26*. (A)** The levels of triterpenes after co-expressing *NaOSC2* with *NaCYP716E107* or *SlCYP716E26*. **(B)**The levels of triterpenes in *NaCYP716E107* or *SlCYP716E26* expressed *N. benthamiana* leaves with erythrodiol (N2), oleanolic acid (N4), or maslinic acid (N7) as substrates. N1: β-amyrin, N2: erythrodiol, N4: oleanolic acid, N6: daturadiol, N7: maslinic acid, N9: putative 6β-hydroxy oleanolic acid. ANOVAs with Tukey's test and independent Samples Kruskal-Wallis test were used for statistical analyses with a significance level of 0.05 (n=3, mean ± SE, different letters indicate significant differences between the two groups at the 0.05 level). The central line within the box represents the median of the data. The upper and lower boundaries of the box denote the upper and lower quartiles of the data. The lines above and below the box, known as whiskers, signify the variability of the data (error bar). The whiskers’ length is set at 1.5 times the interquartile range. The points represent specific observations in the data set. The values beyond 1.5 times the interquartile range are considered as outliers.





**Supplementary Figure** **S17 Silencing efficiency of 5 triterpene biosynthesis genes in VIGS plants.** EV: empty vector plants. Results of two-tailed Student's *t*-test are shown (n= 4~6 replicate plants, mean ± SE; *, *p*< 0.05; **, *p* < 0.01; ***, *p* < 0.001; ****, *p* < 0.0001). The central line within the box represents the median of the data. The upper and lower boundaries of the box denote the upper and lower quartiles of the data. The lines above and below the box, known as whiskers, signify the variability of the data (error bar). The whiskers’ length is set at 1.5 times the interquartile range. The points represent specific observations in the data set. The values beyond 1.5 times the interquartile range are considered as outliers.


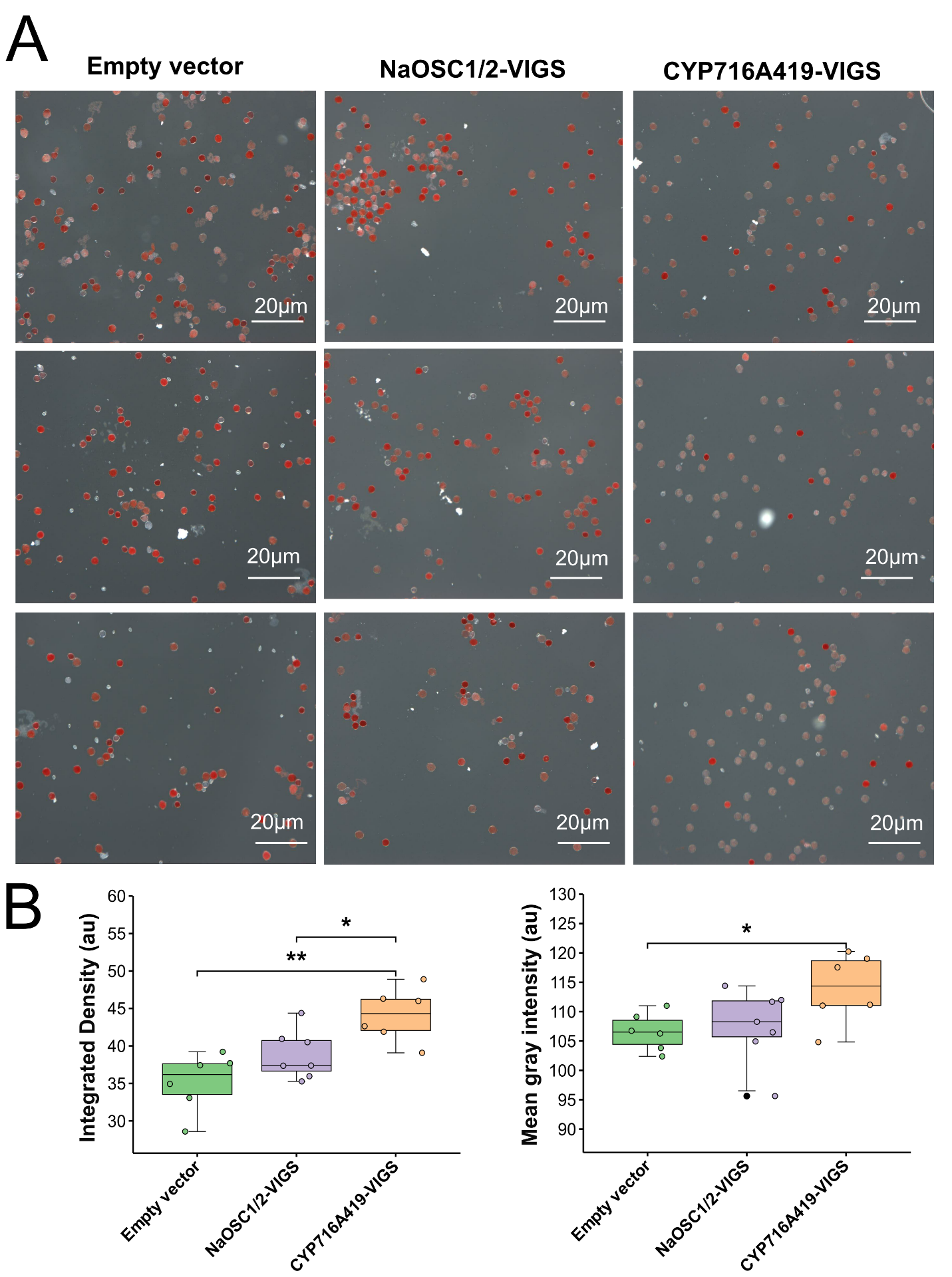


**Supplementary Figure S18 2,3,5-triphenyltetrazolium chloride (TTC) staining of pollen from EV (empty vector), NaOSC1/2-VIGS, and CYP716A419-VIGS plants. (A)** TTC staining of pollen. **(B)** the integrated density and mean gray intensity of stained pollen. Red-stained pollen reflect viable pollen. Each image is of similar numbers of pollen grains from 3 replicate plants (pooled from 5 stamens/flowers). Three different fields of view were randomly selected for each repetition. Utilize Fiji (ImageJ) to process each image and compute the average integrated density (IntDen) and mean gray intensity (mean) of all pollen in each image. Integrated density denotes the cumulative gray values within the actual area of the region of interest (ROI), while mean gray intensity signifies the average gray value across the entire ROI. 【IntDen】= 【Mean】 x 【Area_Size】. Results of two-tailed Student's *t*-test are shown (n=6~8, mean ± SE; *, *p*< 0.05; **, *p* < 0.01; ***, *p* < 0.001; ****, *p* < 0.0001). The central line within the box represents the median of the data. The upper and lower boundaries of the box denote the upper and lower quartiles of the data. The lines above and below the box, known as whiskers, signify the variability of the data (error bar). The whiskers’ length is set at 1.5 times the interquartile range. The points represent specific observations in the data set. The values beyond 1.5 times the interquartile range are considered as outliers.





**Supplementary Figure S19** **GC-MS chromatograms of extracts of *N. attenuata* roots with phytohormone treatments.** The blue-labeled peaks represent known peaks, while the red-labeled ones represent unknown peaks. A6: 28-hydroxy lupanediol, A8: 28-carboxy lupanediol, 28-aldehyde lupanediol.


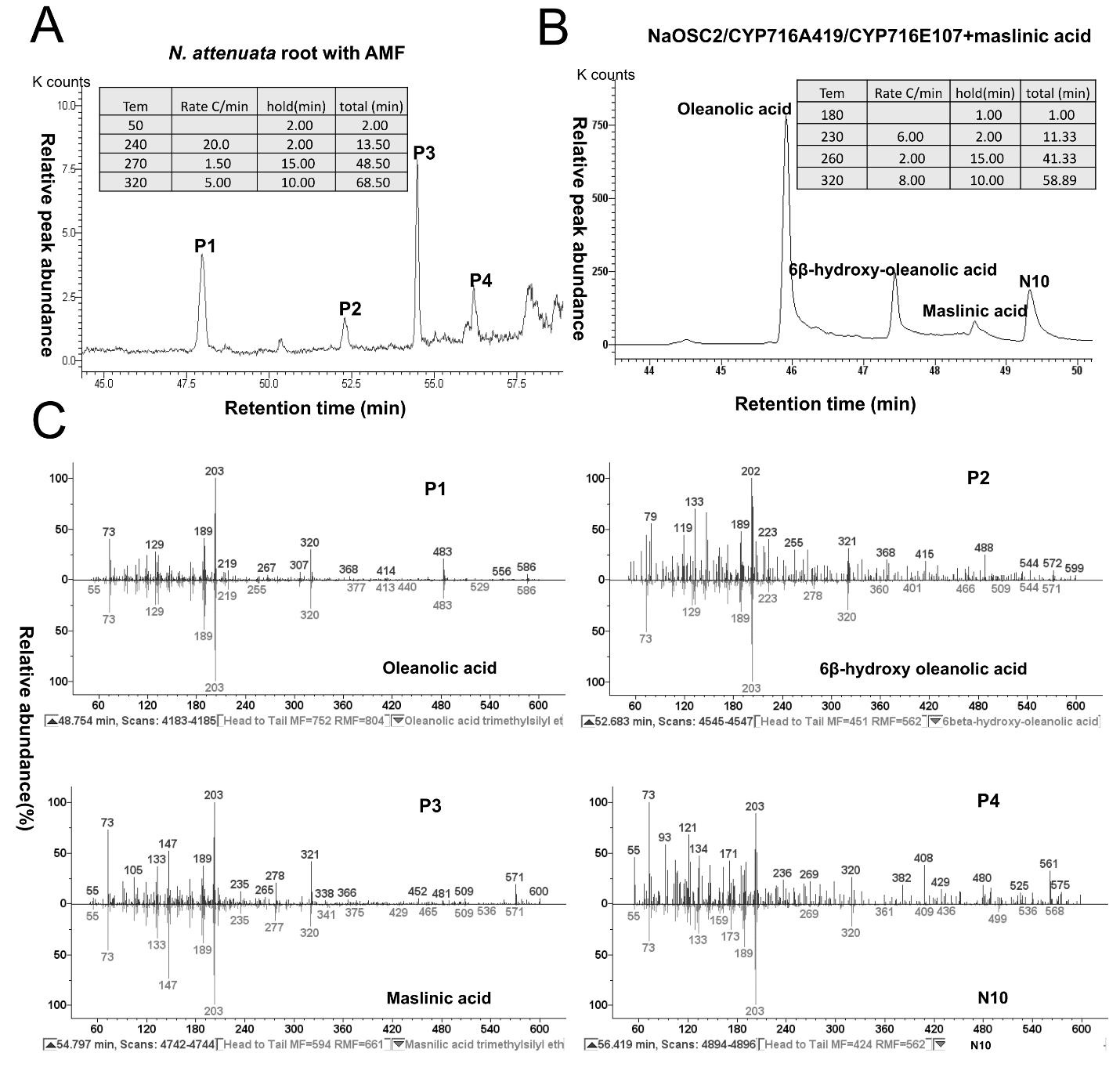


**Supplementary Figure S20 Products of** **NaCYP716A419, NaCYP716C87, and NaCYP716E107 in *N. attenuata* roots (infected with arbuscular mycorrhizal fungi (AMF) for 5 weeks). (A)** GC-MS chromatograms of extracts of *N. attenuata* roots infected with AMF for 5 weeks. **(B)** GC-MS chromatograms of extracts of *N. benthamiana* leaves co-expressed with *NaOSC2/CYP716A419/ CYP716E107* in which maslinic acid standard was added before trimethylsilane derivatization. **(C)** Comparison of EI-MS spectra between the unidentified triterpenes detected in *N. attenuata* roots and the products of CYP716A419/CYP716E107, and a maslinic acid standard. Peak retention times are shifted in panels **(A)** and **(B)** due to runs being conducted with different temperature ramp programs to optimize product separations. The employed temperature ramp profiles are presented in the green and orange tables. All runs were acquired on the same instrument, chromatographic column, and mass spectrometry conditions.


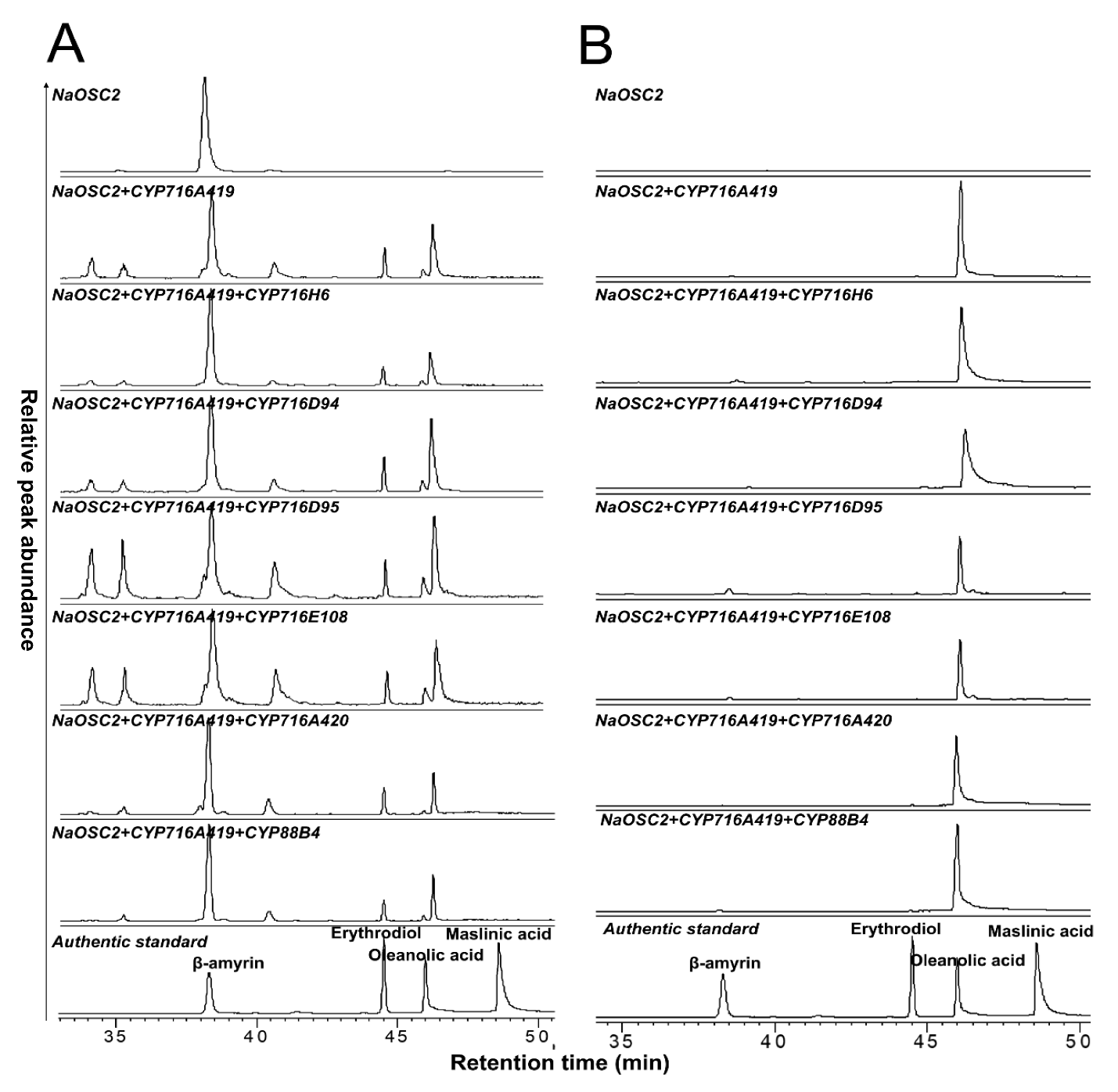


**Supplementary Figure S21 GC-MS chromatograms of reaction products of *NaOSC2/CYP716A419* co-expressed with other CYP450 candidates, aligned with standards. (A)** The enzyme products profile in alkaline extracts of leaves. **(B)** The enzyme products profile in acidic extracts of leaves.


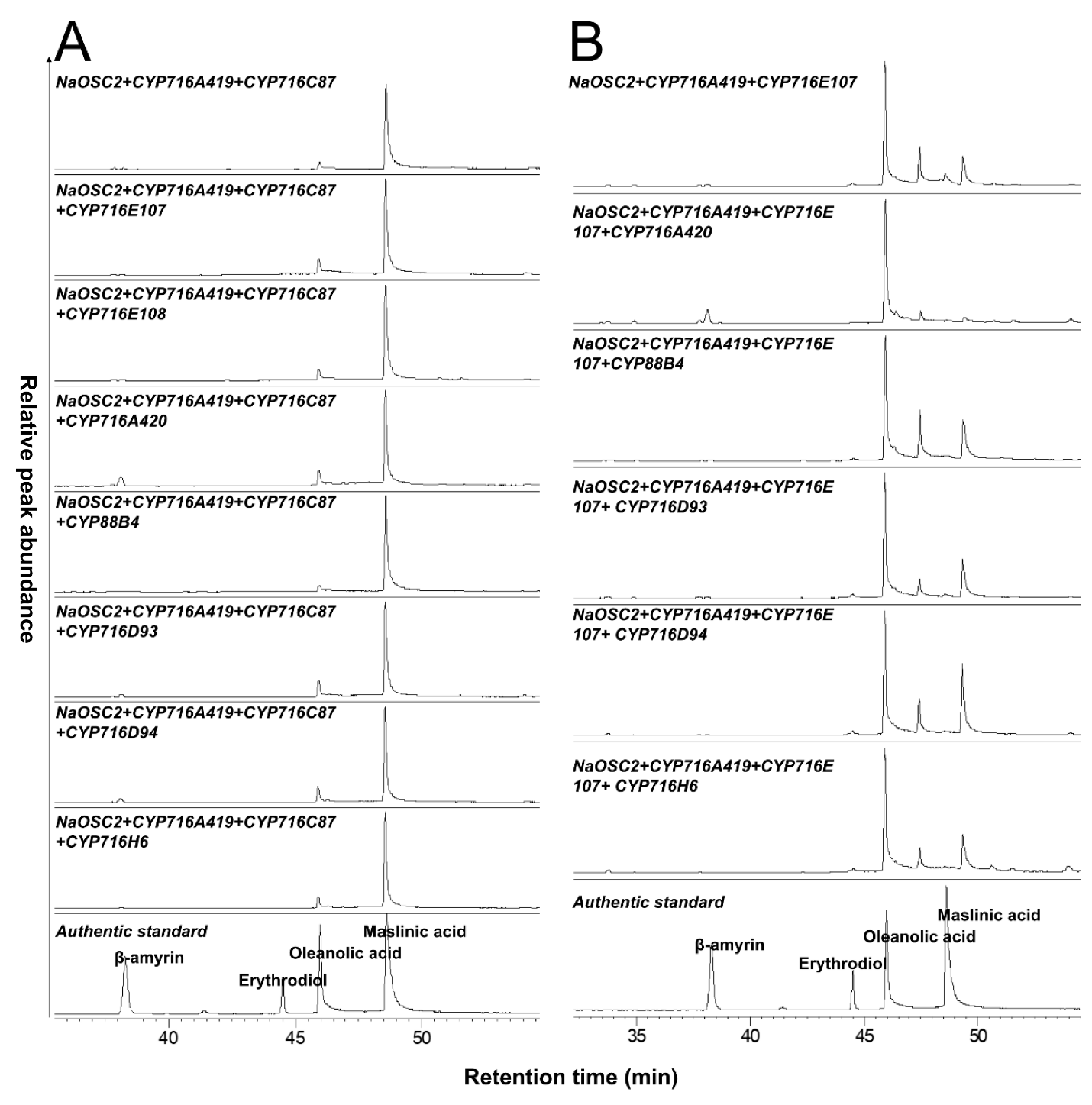


**Supplementary Figure S22 GC-MS chromatograms of reaction products of *NaOSC2/NaCYP716A419/NaCYP716C87* or *NaOSC2/NaCYP716A419/NaCYP716E107* co-expressed with other CYP450 candidates in *N. benthamiana*, aligned with authentic standards. (A)** The enzyme products profile (alkaline + acidic) of leaves expression *NaCYP716C87* and other CYP enzymes. **(B)** The enzyme products profile (alkaline + acidic) of leaves expression *NaCYP716E107* and other CYP enzymes.

**References**

**Brockmöller T, Ling Z, Li D, Gaquerel E, Baldwin IT, Xu S** (2017) *Nicotiana attenuata* Data Hub (NaDH): an integrative platform for exploring genomic, transcriptomic and metabolomic data in wild tobacco. BMC Genomics **18:** 79
