# Supplementary material for: Roles of three cytochrome P450 monooxygenases in triterpene biosynthesis and their potential impact on growth and development
Source: Plant Physiol. 2024 Jul 25;196(2):1407–25. doi: 10.1093/plphys/kiae399 (PMC11444297; doi:10.1093/plphys/kiae399)
Supplement: kiae399_Supplementary_Data [file kiae399_supplementary_data.zip › Supporting information_tableedited.docx]

## Supplemental Information

**Article title:** **Roles of cytochrome P450 monooxygenases in triterpene biosynthesis and their potential impact on growth and development**

Authors: Caiqiong Yang, Halitschke, Sarah E. O'Connor, and Ian T. Baldwin

**Supplementary Table S1** **Cytochrome P450 enzymes used for phylogenetic tree**

| **Name** | **Plant** | **Description** | **Gene number** | **References** |
| --- | --- | --- | --- | --- |
| CYP51H10 | *Avena strigosa* | C16β-hydroxylase;  C12,13 epoxidase | DQ680852 | (Geisler et al., 2013) |
| CYP716A12 | *Medicago truncatula* | C28-oxidase | DQ335781 | (Carelli et al., 2011) |
| CYP716C11 | *Centella asiatica* | C2α-hydroxylase | KU878852 | (Miettinen et al., 2017) |
| CYP716E26 | *Solanum lycopersicum* | C6β-hydroxylase | XM_004241773 | (Yasumoto et al., 2017) |
| CYP716E41 | *Centella asiatica* | C6β-hydroxylase | KU878851 | (Miettinen et al., 2017) |
| CYP72A154 | *Glycyrrhiza uralensis* | C30-oxidase | AB558153 | (Seki et al., 2011) |
| CYP72A67 | *Medicago truncatula* | C2β-hydroxylase | DQ335780 | (Biazzi et al., 2015) |
| CYP72A68v1 | *Medicago truncatula* | C23-oxidase | XM_013608494 | (Fukushima et al., 2013; Biazzi et al., 2015) |
| CYP88D6 | *Glycyrrhiza uralensis* | C11-oxidase | AB433179 | (Seki et al., 2008) |
| CYP93E1 | *Glycine max* | C24-oxidase | AF135485 | (Shibuya et al., 2006; Moses et al., 2014) |

**Supplementary Table S2** **Cytochrome P450 enzymes from *Nicotiana attenuata***

| **CYP name** | **sequence ID** | **subfamily** | **sequence length** |
| --- | --- | --- | --- |
| CYP88B4 | NIATv7_g33874 | CYP88B | 482 |
| CYP88C16 | NIATv7_g06757 | CYP88C | 392 |
| CYP716A419 | NIATv7_g01943 | CYP716A | 480 |
| CYP716A420 | NIATv7_g18201 | CYP716A | 481 |
| CYP716C87 | NIATv7_g07976 | CYP716C | 479 |
| CYP716D93 | NIATv7_g33423 | CYP716D | 469 |
| CYP716D94 | NIATv7_g17429 | CYP716D | 476 |
| CYP716E107 | NIATv7_g15096 | CYP716E | 456 |
| CYP716E108 | NIATv7_g15098 | CYP716E | 455 |
| CYP716H6 | NIATv7_g03976 | CYP716H | 326 |

**Supplementary Table S3 The level of confidence for β-amyrin derivates identification.** N1: β-amyrin, N2: erythrodiol, N3: putative 2α-hydroxy β-amyrin, N4: oleanolic acid, N5: oleanolic aldehyde, N6: daturadiol, N7: maslinic acid, N8: putative 2α-hydroxy erythrodiol, N9: putative 6β-hydroxy oleanolic acid，N10：putative incomplete derived 6β-hydroxy oleanolic acid.

|  | **N1** | **N2** | **N3** | **N4** | **N5** | **N6** | **N7** | **N8** | **N9** | **N10** |
| --- | --- | --- | --- | --- | --- | --- | --- | --- | --- | --- |
| **level 1** | Authentic standard | Authentic standard |  | Authentic standard |  |  | Authentic standard |  |  |  |
| **level 2** |  |  |  |  | based on the function of CYP716A419 and compared N3‘s GCMS spectrum with reference (Misra et al., 2017) | compared with the reference MS of the CYP716E26 products (Yasumoto et al., 2017) |  |  | Compare the elution order and GCMS of N9 with those CYP716E41 products reported in the literature (Miettinen et al., 2017) | Compare the elution order and GCMS of N10 with those CYP716E41 products reported in the literature (Miettinen et al., 2017) |
| **level 3** |  |  | based on the function of CYP716C87 and compared N3‘s GCMS spectrum with β-amyrin and maslinic acid |  |  |  |  | based on the function of CYP716C87 and N8‘s GCMS spectrum with erythrodiol and maslinic acid |  |  |

**Supplementary Table S4 The level of confidence for lupeol and lupanediol derivates identification.** A1: lupeol, A2: putative 2α-hydroxy lupeol, A3: betulin, A4: betulinic acid, A5: 3alpha,20-lupanediol, A6: putative 28-hydroxy lupanediol, A7: putative 2α-hydroxy lupanediol, A8: putative 28-acrboxy lupanediol, A9: putative 28-aldehyde lupanediol. A10: alphitolic acid, A11: putative 2α-hydroxy 28-carboxy lupanediol.

|  | **A1** | **A2** | **A3** | **A4** | **A5** | **A6** | **A7** | **A8** | **A9** | **A10** | **A11** |
| --- | --- | --- | --- | --- | --- | --- | --- | --- | --- | --- | --- |
| **level 1** | Authentic standard |  | Authentic standard | Authentic standard |  |  |  |  |  |  |  |
| **level 2** |  |  |  |  | Based on the reported function of AtLUP1 and the reported mass spectrometry information((Segura et al., 2000; Moses et al., 2012)) | based on the function of CYP716A419 compared with reference (Moses et al., 2012) with A6‘s GCMS spectrum |  | based on the function of CYP716A419 compared with reference (Moses et al., 2012) with A8‘s GCMS spectrum |  |  |  |
| **level 3** |  | based on the function of CYP716C87 and compared A2‘s GCMS spectrum with lupeol and maslinic acid |  |  |  |  | based on the function of CYP716C87 and compared A2‘s GCMS spectrum with betulin and maslinic acid |  | based on the function of CYP716A419 and A9‘s GCMS spectrum with lupenediol | based on the function of CYP716C87 and compared A10‘s GCMS spectrum with betulinic acid and maslinic acid | based on the function of CYP716C87 and compared A10‘s GCMS spectrum with lupanediol, betulinic acid and maslinic acid |

**Supplementary Table S5** **Primer sequences used for the design of constructs for transient expression**

| **Name** | **Sequence 5’ to 3’** | |
| --- | --- | --- |
| CYP716H6-3Ω1-F | | TTTATGAATTTTGCAGCTCGATGGACAATATGTCACATTTTCAC |
| CYP716H6-3Ω1-R | | GACAACCACAACAAGCACCGTTACAGATTATAAAGGGTAACGGGAT |
| CYP88C16-3Ω1-F | | TTTATGAATTTTGCAGCTCGATGGAGTACGACTTAGTGTTCT |
| CYP88C16-3Ω1-R | | GACAACCACAACAAGCACCGTCATCCCTTGGGTATGGCGTACC |
| CYP716E107-3Ω1-F | | TTTATGAATTTTGCAGCTCGATGGAAGCCATGATTTCTTACCT |
| CYP716E107-3Ω1-R | | GACAACCACAACAAGCACCGTTAATTTTCATGAGGCAGAAGACGA |
| CYP716D94-3Ω1-F | | TTTATGAATTTTGCAGCTCGATGTCAATGGAAATAGTAATTAGC |
| CYP716D94-3Ω1-R | | GACAACCACAACAAGCACCGTTAAGGGCTGTGAGGTTGAA |
| CYP716A420-3Ω1-F | | TTTATGAATTTTGCAGCTCGATGGAGTTGTTCTATGTCTCTCT |
| CYP716A420-3Ω1-R | | GACAACCACAACAAGCACCGTCATGCCTTGTTGTGAGGATA |
| CYP716D93-3Ω1-F | | TTTATGAATTTTGCAGCTCGATGGCTACAATTAACTTCCCATCTG |
| CYP716D93-3Ω1-R | | GACAACCACAACAAGCACCGTCAAGGCTTATGAGTTTGAAGGCTA |
| CYP88B4-3Ω1-F | | TTTATGAATTTTGCAGCTCGATGGAGTACTACAATTTAGCTTTCT |
| CYP88B4-3Ω1-R | | GACAACCACAACAAGCACCGCTAGGCTGAGAGCTTCTTGAATCT |
| CYP716E107-3Ω1-F | | TTTATGAATTTTGCAGCTCGATGGAAGCCATGATTTCTTACCTAT |
| CYP716E107-3Ω1-R | | GACAACCACAACAAGCACCGTTAATTTTCATGAGGCAGAAGACGAAC |
| CYP716A419-3Ω1-F | | TTTATGAATTTTGCAGCTCGATGGAGGTCTTCTACCTCTATCTCCTTTGC |
| CYP716A419-3Ω1-R | | GACAACCACAACAAGCACCGCTAAGCTTTGTGAGGATAAAGTCTAACAGG |
| CYP716C87-3Ω1-F | | TTTATGAATTTTGCAGCTCGATGGATTCTTTTGATCCCTCCGCCCTTGTCGTT |
| CYP716C87-3Ω1-R | | GACAACCACAACAAGCACCGTTAATGTTGGTGAAGACGGATAGGAAGTCC |
| CYP88C16-3Ω1-F | | TTTATGAATTTTGCAGCTCGATGGAGTACGACTTAGTGTTCTTGTACACAG |
| CYP88C16-3Ω1-R | | GACAACCACAACAAGCACCGTCATCCCTTGGGTATGGCGTACCCATTTATG |
| CYP716E26-3Ω1-F | | TTTATGAATTTTGCAGCTCGATGGATCCCTTTATTCTTTATTCACTAGC |
| CYP716E26-3Ω1-R | | GACAACCACAACAAGCACCGTCAAGCTATGGGTTGAATCCTAACAGGGAG |
| NaOSC2-3Ω1-F | | TTTATGAATTTTGCAGCTCGATGTGGAAGTTGAAGATTGCAGAA |
| NaOSC2-3Ω1-R | | GACAACCACAACAAGCACCGTTAGTTGTTTTGTAATGGTGATAGG |
| AtLUP1-3Ω1-F2 | | TTTATGAATTTTGCAGCTCGATGTGGAAGTTGAAGATAGGAAAGGGAAATG |
| AtLUP1-3Ω1-R2 | | GACAACCACAACAAGCACCGTTAATTAACGATAAACACAACTTTTCGGTA |

**Supplementary Table S6.** **Primer sequences for VIGS**

| **Name** | **Sequence 5’ to 3’** |
| --- | --- |
| CYP716E107-SalI-5‘UTR-F | GCGGCGGGTCGACAAATAAAATATTGATATCATATGCTTTTC |
| CYP716E107-BamHI-5‘UTR-R | GCGGCGGGATCCTTCTTTCTCTTCCTTGTTTGCTATGTAT |
| CYP716C87-SalI-CDS-F | GCGGCGGTCGACATGGATTCTTTTGATCCCTCCGCCCTTG |
| CYP716C87-BamHI-CDS-R | GCGGCGGGATCCAGTAAAGAGTTTCTCTTCGTTTGAGAA |
| CYP716A419-SalI-5‘UTR-F | GCGGCGGGTCGACTAATTAAATAACTTTAATGTTTCTTAAAATATCT |
| CYP716A419-BamHI-5’UTR-R | GCGGCGGGATCCGTTTAGTTTTGTGGTTAAGGAAATAAGAAAATC |
| NaOSC1/2-SalI-CDS-F | TCAGTCGACCGTTGGTTCAAGATTTGATGTGGGACAGT |
| NaOSC1/2-BamHI-CDS-R | AGTCAGGATCCCATTCTTGACTACCAAAACTCTGCA |

**Supplementary Table S7.** **Primer sequences for qPCR**

| **Name** | **Sequence 5’ to 3’** |
| --- | --- |
| NaCYP716A419-F | CTTGACCGGAACCCTACCTC |
| NaCYP716A419-R | TTCAGGGTGACCTTTCCAACC |
| NaCYP716E107-F | ATGGCTGTGTTTTGTGGTGC |
| NaCYP716E107-R | GGGCCAAACAATCCATAACTGG |
| NaCYP716C87-F | CCCCAAGGGTTGGAAGGTTT |
| NaCYP716C87-R | GAATTGGCTCGTCGCGTTTC |
| NaOSC1-F | CTAGCTCGTCAATCCAAGCA |
| NaOSC1-R | AAGCACACACCCCAGTTACC |
| NaOSC2-F | TGGCTGAATACCGCAAAAATGTCC |
| NaOSC2-R | TTGGGGGTAAACAGTAAGGACCAA |
| IF5a-F | GTCGGACGAAGAACACCATT |
| IF5a-R | CACATCACAGTTGTGGGAGG |

**References**

**Biazzi E, Carelli M, Tava A, Abbruscato P, Losini I, Avato P, Scotti C, Calderini O** (2015) CYP72A67 catalyzes a key oxidative step in medicago truncatula hemolytic saponin biosynthesis. Molecular Plant **8:** 1493-1506

**Carelli M, Biazzi E, Panara F, Tava A, Scaramelli L, Porceddu A, Graham N, Odoardi M, Piano E, Arcioni S, May S, Scotti C, Calderini O** (2011) *Medicago truncatula* CYP716A12 is a multifunctional oxidase involved in the biosynthesis of hemolytic saponins. The Plant Cell **23:** 3070-3081

**Fukushima EO, Seki H, Sawai S, Suzuki M, Ohyama K, Saito K, Muranaka T** (2013) Combinatorial biosynthesis of legume natural and rare triterpenoids in engineered yeast. Plant and Cell Physiology **54:** 740-749

**Geisler K, Hughes RK, Sainsbury F, Lomonossoff GP, Rejzek M, Fairhurst S, Olsen CE, Motawia MS, Melton RE, Hemmings AM, Bak S, Osbourn A** (2013) Biochemical analysis of a multifunctional cytochrome P450 (CYP51) enzyme required for synthesis of antimicrobial triterpenes in plants. Proc Natl Acad Sci U S A **110:** E3360-3367

**Miettinen K, Pollier J, Buyst D, Arendt P, Csuk R, Sommerwerk S, Moses T, Mertens J, Sonawane PD, Pauwels L, Aharoni A, Martins J, Nelson DR, Goossens A** (2017) The ancient CYP716 family is a major contributor to the diversification of eudicot triterpenoid biosynthesis. Nature Communications **8:** 14153

**Misra RC, Sharma S, Sandeep, Garg A, Chanotiya CS, Ghosh S** (2017) Two CYP716A subfamily cytochrome P450 monooxygenases of sweet basil play similar but nonredundant roles in ursane- and oleanane-type pentacyclic triterpene biosynthesis. New Phytologist **214:** 706-720

**Moses T, Goossens Ap, Thevelein Jc** (2012) Metabolic engineering for production of triterpenoid saponin building blocks in plants and yeast. 2012.

**Moses T, Thevelein JM, Goossens A, Pollier J** (2014) Comparative analysis of CYP93E proteins for improved microbial synthesis of plant triterpenoids. Phytochemistry **108:** 47-56

**Segura MJR, Meyer MM, Matsuda SPT** (2000) *Arabidopsis thaliana* LUP1 converts oxidosqualene to multiple triterpene alcohols and a triterpene diol. Organic Letters **2:** 2257-2259

**Seki H, Ohyama K, Sawai S, Mizutani M, Ohnishi T, Sudo H, Akashi T, Aoki T, Saito K, Muranaka T** (2008) Licorice β-amyrin 11-oxidase, a cytochrome P450 with a key role in the biosynthesis of the triterpene sweetener glycyrrhizin. Proceedings of the National Academy of Sciences **105:** 14204-14209

**Seki H, Sawai S, Ohyama K, Mizutani M, Ohnishi T, Sudo H, Fukushima EO, Akashi T, Aoki T, Saito K, Muranaka T** (2011) Triterpene functional genomics in licorice for identification of CYP72A154 involved in the biosynthesis of glycyrrhizin     The Plant Cell **23:** 4112-4123

**Shibuya M, Hoshino M, Katsube Y, Hayashi H, Kushiro T, Ebizuka Y** (2006) Identification of β-amyrin and sophoradiol 24-hydroxylase by expressed sequence tag mining and functional expression assay. The FEBS Journal **273:** 948-959

**Yasumoto S, Seki H, Shimizu Y, Fukushima EO, Muranaka T** (2017) Functional characterization of CYP716 family P450 enzymes in triterpenoid biosynthesis in tomato. Frontiers in Plant Science **8:** 21
