# Supplementary material for: Roles of three cytochrome P450 monooxygenases in triterpene biosynthesis and their potential impact on growth and development
Source: Plant Physiol. 2024 Jul 25;196(2):1407–25. doi: 10.1093/plphys/kiae399 (PMC11444297; doi:10.1093/plphys/kiae399)
Supplement: kiae399_Supplementary_Data [file pp.00711.2024-s06.pdf › pp.00711.2024-s06.pdf]

Plant Physiology®

Roles of Three CYP716 Enzymes in Triterpenes Biosynthesis and Their Potential Impact on Growth and Development

Caiqiong Yang, Rayko Halitschke, Sarah O'Connor, and Ian Baldwin

Review Timeline:

Submission Date:	13-Mar-2024
Editorial Decision:	03-Apr-2024
Resubmission Received:	10-May-2024
Editorial Decision:	01-Jun-2024
Revision Received:	18-Jun-2024
Accepted:	06-Jul-2024

02-Apr-2024

Dr. Ian T Baldwin
Max-Planck-Institut fur chemische Okologie
Department of Molecular Ecology, Hans Knoll strasse 8
Jena D-07745
Germany

RE: CYP716 enzymes enhance triterpene diversity in Nicotiana attenuata; their loss impedes normal growth and development

Dear Dr. Ian Baldwin:

Thank you for giving us the opportunity to review your manuscript for publication in the Plant Physiology. The reviewers have found the manuscript of interest, but have raised a number of concerns that will require your attention. As these concerns will entail substantial changes to the manuscript, you are likely to need additional time to address them. I must therefore reject the manuscript in its present form. Nonetheless, if you are able to address the points raised in review, I would welcome your resubmission of a manuscript that incorporates your responses.
The reviews highlight areas that need to be addressed for your consideration. Please see the detailed comments below.

Please note that any resubmission will be treated as a new entry to the system and is likely to go out for review.

If you do have any queries or concerns, you are welcome to contact me in the first instance.

Thank you again for allowing Plant Physiology to review your work. We look forward to hearing from you soon.

Sincerely,

Editor
Plant Physiology

-- Reviewer comments:
Reviewer #1 (Comments for the Author):

The manuscript reports the discovery of functional P450s （CYP716 family）in triterpene biosynthesis from Nicotianna Attenuata and their functions in plant growth and development. Heterologous transient expression in Nicotianna benthamiana and VIGS were exploited to validate the functions of the P450s. The responses of the functional P450s to various hormonal treatments were also tested. Overall, this work reveals the biochemical functions of the P450s in Nicotianna Attenuata triterpene biosynthesis and growth and development. This work seems technically fine. Some suggestions for improvement are listed below:

1. The title of the manuscript contains a semicolon and seems to be two separate parts. Perhaps change the title to "CYP716 enzymes mediate triterpene diversification and growth and development of Nicotiana attenuata"
2. For those enzymatic products that were identified merely based on GCMS spectra, the structure should be given in a bracket to clearly indicate the level of confidence. It would be good to include a table detailing all the compounds identified and the approaches for identification.
3. Are all the P450s from Nicotianna attenuate? It would be good if abbreviated species names are included, e.g. NtCYP716.
4. Fig.1A, what do the different colors of CYPs stand for? This needs to be explained in the legend. It's important to show the expression patterns of the functional CYP716s identified in this work. This is not apparent in the current presentation.
5. There are many peaks, chemical structures and names presented in the manuscript, yet they seemed not to associate well. It's essential to include the numeric labeling of peaks after the compound names. This makes it easier to read.
6. How many VIGS plants were used for chemical and phenotypical analysis? This needs to be clearly indicated in the text and figure legends.
7. The IUPAC names for the compounds can be given in the text rather than in the figures. Generic names can be used for clarity purpose.
8. The authors claimed the P450s are multifunctional, yet the P450s don't seem to be multifunctional as they just act on one site. Therefore, the word "multifunctional" should be removed.

Reviewer #2 (Comments for the Author):

The authors examined the function of several cytochrome P450 genes from Nicotiana attenuata by overexpression in Nicotiana Benthamiana. In this system, they showed that, similar to homologues from other plants, CYP716A419 hydroxylates β-amyrin and lupeol in three consecutive steps forming an aldehyde and a carboxylic acid, that CYP716C87 hydroxylates the AB rings of β-amyrin and lupeol and that CYP716E107 hydroxylates the C6 position of the β-amyrin and oleanolic acid. They then silenced the genes in their native species using VIGS and showed a certain reduction to plants fitness when grown in a competitive setup.
The first part of the study shows the formation of the different compounds from the examined precursors is a well-organized and mostly convincing way. However, the second part, in N. attenuata is far less convincing. The authors also tend to over-interpret their results, present non-significant results as significant and are lacking any statistics for the first part. If there were indeed no replicates and statistical analyses for this part, and this is not just a choice of presentation, I am afraid that the results reported in this part cannot be presented as significant.
For the second part, there are many fast and efficient methods for genome editing today. I do not think a study trying to show the function of a gene can make do with 50% reductions in VIGS. How can we know if there isn't an importance to the exact ratio of the compounds? Especially for a compound like β-amyrin, which the authors themselves mention can have negative effects on cuticular permeability.

Specific comments

The title does not portray the actual findings. The effects of the metabolites were demonstrated only in a heterologous system, and their effects in N. attenuata were examined only under a competitive environment. We can say that their loss reduces competitive success. Also, the expression was never lost. It was somewhat reduced by VIGS. A more fitting title might be Three Nicotiana attenuata CYP716 enzymes enhance triterpene diversity when expressed in Nicotiana benthamiana.

Fig. 1 - is there a control with an empty vector? What about the other peaks in the chromatogram of CTP716A419?

Line 177, 181 - NaOSC2 and AtLup1 should be italicized, Lup1 should be capitalized. This is true throughout the manuscript, where the gene LUP1 is written as Lup1, and many gene names are not italicized.

Line 186 - Are there statistics for the formation of the new peaks? Even if not presented as a bar graph, some p value should be mentioned. These experiments were performed in a plant that produces its own triterpenes. How can we be sure that under certain conditions it does not produce the peaks attributed to the gene expression?

Line 194 - Should mention peak N5 is oleanolic aldehyde.

Lines 210-212 - How can we know that CYP716A419 isn't catalyzing the first step and then native N. benthamiana enzymes are catalyzing the rest? Or maybe they are occurring spontaneously?

Fig. 3A - If there is an empty vector for the "regular" extraction, shouldn't there be one for the acid extraction as well?

Lines 217 - 219 - I think a more full reasoning of how these mass fragments infer that the hydroxylation is on the AB rings is necessary. I managed to understand it, but it took me quite some time and going back and forth with Fig. S5. In the end, I felt the best reasoning for this was that the fragmentation was similar to that of maslinic acid, which we know is hydroxylated at the suggested position.

Fig. S5 - The legend should mention that the suggested modifications by the enzyme are highlighted in green or blue.
The mass of 365 seems to be wrong for the structure drawn at that mass, and should have a mass of 367 (as it gives when drawn in chemdraw). Also, that way losing $OHSi(CH_3)_3$, which has a mass of 90 would bring us from 367 to 277. Losing that mass again from the 277 fragment does not bring us to 189. It brings us to 187, which is really drawn with a loss of an O-TMS and a gain of a hydrogen in its place. Since the identification of the compound relies on the fragmentation patterns, I suggest the authors look into the drawings in Fig. S5 - S7 to ensure they are correct.

This 277 mass fragment also appears in the fragmentation of compounds lacking C2a oxydation, albeit at lower levels, but indicating that this is not a unique fragment. I would therefore rely more on the similarity to the maslinic acid fragmentation. It would also be nice to have the fragmentation of β-amyrin to compare to, so we can see how the peaks are altered.

Line 247 - Lupane and oleanane don't have a hydroxyl on rings A or B, unlike lupeol and oleanol

Fig. 5 Why is the daturadiol peak that is formed with the CYP716E107 so much smaller than the E26 peak?
The N9 compound peak is very small when CYP716A419 isn't expressed. Are there any statistics for this peak? Compared to a control of oleanolic acid? If the direct precursor is oleanolic acid, which seems more abundant in relation to the N9 peak in the samples where CYP716A419 was not expressed, why is this peak so small compared to the ones where it was expressed?

Line 280 - How was the oleanolic acid abundance compared between the treatments? The peaks formed seem too small to affect the abundance of the substrate.

Line 285 - Since peak N10 is not formed with oleanolic acid feeding but is with the CYP716A419, shouldn't N10 be hydroxylated oleanolic aldehyde? Did N10 have the 365 and 189 peaks? Also 2a-hydroxy-β-amyrin didn't have standards but it was inferred based on fragmentation. Was N10 a triterpene? Was it compared to mass fragmentation libraries?

296 - NaCl is not mimicking salt stress, but is actual salt stress.

Lines 309-310 - GA is involved in growth, but is antagonistic to ABA. the fact that there is a fast response to both, in addition to a response to JA in some cases, which also inhibits growth, may indicate a more complex response, and not just a role in "growth and development"?

Line 322 - 324 - Why were these stresses chosen? Why not heat, or light, or lack of fertilization, or bacteria, or insects?

Fig. 7 - the statistical analysis method should be chosen. Either a student's t-test, or a Tukey HSD (or any other method). How are we supposed to treat the difference between EOSC1/2 plants in stalk height and diameter? Is it significant or not?

Lines 383 -385 - The difference between β-amyrin abundances in CYP716A419 plants' theca is not significant. Writing it in the same line with two significant results is misleading. The same is true for lines 385-386 - only one of the three "decreases" in erythrodiol reported is significant, and only two out of three of the oleanolic acid.

Fig. 8 - why was the abundance of olivatolic acid not examined? And that of the 2a hydroxy products? Basically, once there are mutants in the native species of the genes I'd expect to see how silencing these genes affects the metabolites in the native system and not only in a heterologous system.

Line 396 - This is the first time dammarenediol II is mentioned. Why was it searched for?

If none of the downstream metabolites were found, how can we assume that the phenotypes seen in the leaves are related to these missing compounds? Especially when the leaf phenotype exists for both the highest and lowest β-amyrin lines?

Lines 400 -401: How does a reduction of ~50% in OSC expression reduce a metabolite's abundance by 99%? This is not common even for complete CRISPR knock outs.

Line 417 - CYP716C87 affected only dammarenediol II. CYP716E107 affects compounds in 2/11 conditions. The effects on growth were shown only in competition with other plants. saying that "silencing these biosynthetic genes results in perturbations to in both the triterpene content (Fig. 8) and growth of N. attenuata" is a bit of a stretch.

Lines 505-506 - How is responding to every hormone application tight regulation?

Line 509 - The inference was not tested, and the genes weren't silenced, but somewhat reduced.

Line 515 - Was leaf chlorosis quantified? Either by chlorophyl extraction or spectroscopic methods? If not, how can this be stated as a fact?

Lines - 522 - 524 - osc mutants have extremely low β-amyrin compared to control, whereas ctp716a419 mutants have slightly higher β-amyrin. I would expect the EV to have intermediate values between the two. Why are the osc the intermidiates?

Lines 575 - 578 - if the reason for the phenotypes is autotoxicity, why are competitive conditions necessary to reveal them?

Lines 579 - 580 - this paper does not elucidate the biochemical functions in N. attenuata, but elucidates the biochemical functions of N. attenuata genes in N. benthamiana.

Lines 582 - 583 - The roles of these genes were elucidated in N. benthamiana and not in N. attenuata.

Line 584 - nothing was revealed about detoxification by these enzymes. It was suggested along with other equally valid suggestions.

Lines 611 - 612 - Why was the hormonal response profiling performed on the roots? When all the phenotypes and metabolite quantification are performed in the shoot.

Line 631 - why is the 3Ω1 vector cited once for Cárdenas et al., and once for Hong et al., who cites Cárdenas?

Lines 716 - 723 - the statistical analysis section is missing the part of the student's t-tests, which were used in figure 8. Tukey HSD was also not used in Fig. 6A. S8 and S9.
However, most concerningly, no statistics whatsoever seem to have been performed for the first section of the paper. Were they performed and not mentioned?

Fig. S10 -This figure seems out of place. Mentioning a result for the first time in the discussion section and heavily relying on a previous publication. Maybe this could be further developed in the results? Or removed altogether?

Manuscript ID: PP2024-RA-00396

Title: *CYP716 enzymes enhance triterpene diversity in Nicotiana attenuata; their loss impedes normal growth and development*

Dear Editors and Reviewers,

Thank you for your letter and the reviewers' comments on our manuscript. We sincerely appreciate the valuable feedback provided by the reviewers regarding our manuscript with the ID: PP2024-RA-00396. By addressing these insightful comments, the quality of our manuscript has been significantly enhanced. We very much appreciate the editors and reviewers' intellectual generosity.

We made the following major modifications to the manuscript:

1. Change the title to "Three cytochrome P450 from *Nicotiana attenuata* play key roles in triterpene biosynthesis"
2. Added new Tables S6 and S7 that describe the level of confidence for the identifications of enzymatic products.
3. Rewrote the Results and Discussion sections.
4. Added new data for the qPCR detection of NaCYP716A419, NaCYP716C87, and NaCYP716E107 genes in roots, leaves, and flowers.
5. Redrew Figs.1-7.
6. Added new data on the detection of enzyme products of NaCYP716A419, NaCYP716C87, and NaCYP716E107 in *N. attenuata* roots after elicitation by different hormones.
7. Supplemented statistical analysis results of enzyme products obtained after heterologous expression in *N. benthamiana*.

In the following, we proide a point-by-point responses to the reviewers' comments:

Reviewer #1

The manuscript reports the discovery of functional P450s （CYP716 family）in triterpene biosynthesis from Nicotianna Attenuata and their functions in plant growth and development. Heterologous transient expression in Nicotianna benthamiana and VIGS were exploited to validate the functions of the P450s. The responses of the functional P450s to various hormonal treatments were also tested. Overall, this work reveals the biochemical functions of the P450s in Nicotianna Attenuata triterpene biosynthesis and growth and development. This work seems technically fine. Some suggestions for improvement are listed below:

1. The title of the manuscript contains a semicolon and seems to be two separate parts. Perhaps change the title to "CYP716 enzymes mediate triterpene diversification and growth and development of Nicotiana attenuata"

   Response: Thank you so much for the suggestion. we changed the manuscript title to "Three cytochrome P450 from *Nicotiana attenuata* play key roles in triterpene biosynthesis"

2. For those enzymatic products that were identified merely based on GCMS spectra, the structure should be given in a bracket to clearly indicate the level of confidence. It would be good to include a table detailing all the compounds identified and the approaches for identification.

   Response: A great suggestion. We add two tables that provide the level of confidence and approaches for enzymatic product identification. Please refer to the details in **Table S3 and Table S4**.

3. Are all the P450s from Nicotianna attenuate? It would be good if abbreviated species names are included, e.g. NtCYP716.
   Response: Thanks for this suggestion. we add abbreviated species names before the P450s enzyme's name.

4. Fig.1A, what do the different colors of CYPs stand for? This needs to be explained in the legend. It's important to show the expression patterns of the functional CYP716s identified in this work. This is not apparent in the current presentation.

   Response: We explained the meanings of different colors of CYPs in the legend and presented the expression patterns in different tissues of the functional CYP716s in **Fig. 1A**'s heatmap. We have redesigned the figure to make the color codes easier to see and have added text to the legend. For the expression patterns of the CYP716s of this paper, please refer to NIATv7_g01943(CYP716A419), NIATv7_g15096(CYP716E107), and NIATv7_g07976(CYP716C87) in **Fig. 1A**. We also added new qPCR results for different plant tissues in **Fig. 6A**.

5. There are many peaks, chemical structures and names presented in the manuscript, yet they seemed not to associate well. It's essential to include the numeric labeling of peaks after the compound names. This makes it easier to read.

   Response: We include the numeric labeling of peaks after the compound names in the text. These labels are now used consistently throughout the manuscript.

6. How many VIGS plants were used for chemical and phenotypical analysis? This needs to be clearly indicated in the text and figure legends.
   Response: We included the number of replicate VIGS plantsused for chemical and phenotypic analysis in the text and figure legends. Please refer to the legends of **Fig. 7** and **Fig. 8** for details.

7. The IUPAC names for the compounds can be given in the text rather than in the figures. Generic names can be used for clarity purpose.
   Response: Thanks for the suggestion. we changed the IUPAC names of compounds to generic names in the figures.

8. The authors claimed the P450s are multifunctional, yet the P450s don't seem to be multifunctional as they just act on one site. Therefore, the word "multifunctional" should be removed.
   Response: Thanks for the suggestion. we removed the word "multifunctional" for these P450s.

Reviewer #2

The authors examined the function of several cytochrome P450 genes from Nicotiana attenuata by overexpression in Nicotiana Benthamiana. In this system, they showed that, similar to homologues from other plants, CYP716A419 hydroxylates β-amyrin and lupeol in three consecutive steps forming an aldehyde and a carboxylic acid, that CYP716C87 hydroxylates the AB rings of β-amyrin and lupeol and that CYP716E107 hydroxylates the C6[illegible] position of the β-amyrin and oleanolic acid. They then silenced the genes in their native species using VIGS and showed a certain reduction to plants fitness when grown in a competitive setup.

The first part of the study shows the formation of the different compounds from the examined precursors is a well-organized and mostly convincing way. However, the second part, in N. attenuata is far less convincing. The authors also tend to over-interpret their results, present non-significant results as significant and are lacking any statistics for the first part. If there were indeed no replicates and statistical analyses for this part, and this is not just a choice of presentation, I am afraid that the results reported in this part cannot be presented as significant.

For the second part, there are many fast and efficient methods for genome editing today. I do not think a study trying to show the function of a gene can make do with 50% reductions in VIGS. How can we know if there isn't an importance to the exact ratio of

the compounds? Especially for a compound like β-amyrin, which the authors themselves mention can have negative effects on cuticular permeability.

Specific comments

1. The title does not portray the actual findings. The effects of the metabolites were demonstrated only in a heterologous system, and their effects in N. attenuata were examined only under a competitive environment. We can say that their loss reduces competitive success. Also, the expression was never lost. It was somewhat reduced by VIGS. A more fitting title might be Three Nicotiana attenuata CYP716 enzymes enhance triterpene diversity when expressed in Nicotiana benthamiana.
   Response: Thanks for the suggestion. we change the title to "Three cytochrome P450 from *Nicotiana attenuata* play key roles in triterpene biosynthesis"

2. Fig. 1 - is there a control with an empty vector? What about the other peaks in the chromatogram of CTP716A419?
   Response: Thanks for this important point. We have now added in **Fig. 1C** a control with an empty vector. The statistical results of the detected peaks of triterpenes in **Fig. 1** are now detailed in **Fig. S3**. Additionally, co-expression of the empty vector (EV) with NaCYP716A419, instead of NaOSC2 (providing β-amyrin), did not yield erythrodiol (N2), oleanolic aldehyde, or oleanolic acid (**Fig. S4A**). In the leaves expressing the empty vector, the addition of erythrodiol also did not yield oleanolic aldehyde, and oleanolic acid (**Fig. S4B**).

3. Line 177, 181 - NaOSC2 and AtLup1 should be italicized, Lup1 should be capitalized. This is true throughout the manuscript, where the gene LUP1 is written as Lup1, and many gene names are not italicized.
   Response: Thanks for these points. The non- italicized NaOSC2 and AtLup1 in the text indicate the enzyme name. To more clearly distinguish the enzyme and gene names in the revision, we use of NaOSC2 and AtLPU1 to represent the enzyme name and use *Naosc2* and *Atlup1* to represent the gene names.

4. Line 186 - Are there statistics for the formation of the new peaks? Even if not presented as a bar graph, some p value should be mentioned. These experiments were performed in a plant that produces its own triterpenes. How can we be sure that under certain conditions it does not produce the peaks attributed to the gene expression?
   Response: We added statistics for the formation of the new peaks: please refer to **Fig. S3** for details. As shown in the reply to question No. 2 above, in the empty vector controls in *N. benthamiana* β -amryin is only found in trace amounts. Expressing NaCYP716A419 (see **answer to question No. 2**), NaCYP716C87 (see **Fig. 3**), or NaCYP716E107 (see **Fig. 5**) in *N. benthamiana* without the addition of substrates, did not produce these peaks. Moreover, in **Fig. 1C and Fig. S3**, when plants express NaOSC2 and other CYP enzymes, no new peaks apart from β-amyrin were observed. Taken together, these results suggest that empty vectors, NaOSC2, or non-functional

CYPs do not elicit endogenous expression under our experimental conditions.

5. Line 194 - Should mention peak N5 is oleanolic aldehyde.
   Response: Thanks for this suggestion; we added the names of the compounds.

6. Lines 210-212 - How can we know that CYP716A419 isn't catalyzing the first step and then native N. benthamiana enzymes are catalyzing the rest? Or maybe they are occurring spontaneously?
   Response: Thanks for this important point. We deleted the statement of "consecutive three-step", but note that there are several CYP716A enzymes that show this three-step oxidation function (Carelli et al., 2011; Fukushima et al., 2011; Miettinen et al., 2017; Misra et al., 2017).

7. Fig. 3A - If there is an empty vector for the "regular" extraction, shouldn't there be one for the acid extraction as well?
   Response: Thanks for this important point. We added chromatograms of base and acid extractions from plants expressing the empty vector and separated them into Panel A and Panel B in the revised **Fig. 3**.

8. Lines 217 - 219 - I think a more full reasoning of how these mass fragments infer that the hydroxylation is on the AB rings is necessary. I managed to understand it, but it took me quite some time and going back and forth with Fig. S5. In the end, I felt the best reasoning for this was that the fragmentation was similar to that of maslinic acid, which we know is hydroxylated at the suggested position.
   Response: Thanks for this important point, which we incorporate into the revision. See highlighed text in the results section part_3 (NaCYP716C87 is a C2α oxidase).

9. Fig. S5 - The legend should mention that the suggested modifications by the enzyme are highlighted in green or blue.
   Response: The colors are now added to the Figure.

10. The mass of 365 seems to be wrong for the structure drawn at that mass and should have a mass of 367 (as it gives when drawn in ChemDraw). Also, that way losing OHSi(CH3)3, which has a mass of 90 would bring us from 367 to 277. Losing that mass again from the 277 fragment does not bring us to 189. It brings us to 187, which is drawn with a loss of an O-TMS and a gain of a hydrogen in its place. Since the identification of the compound relies on the fragmentation patterns, I suggest the authors look into the drawings in Fig. S5 - S7 to ensure they are correct.
    Response: Thanks for thoughtful comments. We recalculated and redrew the fragmentation pathways of these structures and revised the main text accordingly and added several new supplemental Figures (S7-S15).

    The derivatization pathway of the β-amyrin-TMS standard, after undergoing EI-MS fragmentation, is illustrated in **Fig. S7**. β-amyrin-TMS undergoes retro-Diels–Alder

cleavage (RDA), resulting in m/z 218 and m/z 279. m/z 218 loses a methyl group and gains an electron to yield m/z 203, while m/z 279 loses a TMSOH and forms a double bond to yield m/z 189. When the C2α position is substituted with a hydroxyl group, as in maslinic acid (**Fig. S11**), its trimethylsilylated derivative undergoes a RDA reaction to yield m/z 320 and m/z 367. m/z 320 loses TMSCOOH and gains a proton to yield m/z 203, while m/z 367 looses a TMSOH and forms a double bond to yield m/z 277. Furthermore, m/z 277 continues to loose three methyl groups and gain three protons to yield m/z 235. N3 exhibits m/z 218, 203, and 189, which is similar to the fragmentation pattern of β-amyrin, and simultaneously m/z 277 and 235, matching the steps of AB ring loss ions after RDA cleavage of maslinic acid containing a hydroxyl group at the C2α position. By comparing their GCMS spectra and fragmentation pathways with those of β-amyrin and maslinic acid, N3 was inferred to be the C2α hydroxylated product of β-amyrin, namely 2α-hydroxyl-β-amyrin.

11. This 277 mass fragment also appears in the fragmentation of compounds lacking C2a oxidation, albeit at lower levels, but indicating that this is not a unique fragment. I would therefore rely more on the similarity to the maslinic acid fragmentation. It would also be nice to have the fragmentation of β-amyrin to compare to, so we can see how the peaks are altered.
Response: We have reworked the main text and integrated this point in comparisons of the EI-MS spectra of β-amyrin and maslinic acid. Please refer to the Results section of the main text for the details.

12. Line 247 - Lupane and oleanane don't have a hydroxyl on rings A or B, unlike lupeol and oleanol.
Response: correct!.

13. Fig. 5 Why is the daturadiol peak that is formed with the CYP716E107 so much smaller than the E26 peak?
Response: This is an important observation for which we have a hypothesis. NaCYP716E107 and SlCYP716E26 are enzymes from different plants and are not identical. NaCYP716E107 is our candidate gene from *N. attenuata*, whereas SlCYP716E26 is corresponding C-6β oxidase characterized by Yasumoto et al. (Yasumoto et al., 2017). These authors demonstrated that SlCYP716E26 catalyzes β-amyrin into daturadiol. Here, we utilize SlCYP716E26 as a reference to ascertain whether our peak N6 corresponds to daturadiol or not. Protein sequence alignment reveals significant differences between NaCYP716E107 and SlCYP716E26, as shown in **Fig. S16**. Specifically, at positions 255 to 261, NaCYP716E107 possesses five additional amino acids compared to SlCYP716E26. So we hypothesize that this variation may impact the protein's spatial structure, potentially leading to differences in the affinity of NaCYP716E107 and SlCYP716E26 towards the β-amyrin substrate.

14. The N9 compound peak is very small when CYP716A419 isn't expressed. Are there any statistics for this peak? Compared to a control of oleanolic acid? If the direct

precursor is oleanolic acid, which seems more abundant in relation to the N9 peak in the samples where CYP716A419 was not expressed, why is this peak so small compared to the ones where it was expressed?

Response: The direct precursor of NaCYP716A419 is not oleanolic acid. Extensive research has demonstrated that members of the CYP716A subfamily catalyze β-amyrin to produce oleanolic acid, potentially generating erythrodiol and oleanolic aldehyde as intermediate products (Carelli et al., 2011; Miettinen et al., 2017; Moses et al., 2015). In Fig. 2, we confirmed that CYP716A419 also exhibits this functionality, yielding oleanolic acid as the final product, as depicted in the figure.

Here, the addition of N9 produced by CYP716A419 is greater than that obtained by directly adding oleanolic acid. We believe this discrepancy may be attributed to the following 3 points:

1. We are uncertain about the efficiency of injecting 1 mL of 100 μM oleanolic acid into plant cells; the majority likely remains on the surface of the plant. However, upon adding NaCYP716A419, it can directly convert the product of NaOSC2, β-amyrin, into oleanolic acid inside the plant cells. This results in the abundant production of oleanolic acid in the plant upon co-expression of NaOSC2 and CYP716A419, which can serve as a direct substrate for oxidation by CYP716E107 to yield N9.

2. OSC and CYP are enzymes that function on the endoplasmic reticulum membrane. The transport of oleanolic acid injected into the extracellular space across the plasma membrane to the endoplasmic reticulum requires time. However, the expression of NaOSC2 and NaCYP716A419 *in vivo* may lead to a rapid production of oleanolic acid. Additionally, we cannot rule out the possibility that the generated oleanolic acid can directly undergo further oxidation by NaCYP716E107 within the endoplasmic reticulum to yield N9.

3. Before injecting the substrate, we allowed the enzyme to be expressed in leaves for 3 days. For both the oleanolic acid+CYP716E107 combination and the NaOSC2+ CYP716A419+CYP716E107 combination, the time point when the enzyme is inoculated into *N. benthamiana* is the same. When injecting oleanolic acid, the enzyme has already started to function. However, for the NaOSC2+CYP716A419+CYP716E107 combination that does not require the addition of oleanolic acid. Hence, in comparison to the oleanolic acid+CYP716E107 combination, the time point for N9 production is much earlier in the NaOSC2+CYP716A419+CYP716E107 combination (by 2-3 days). Sampling was conducted 3 days after the injecting oleanolic acid. In terms of substrate content, substrate utilization, and reaction time, the NaOSC2+CYP716A419+CYP716E107 combination outperforms the oleanolic acid+CYP716E107 combination in terms of the yield of N9 obtained and this difference in performance is likely explained by differences in reaction kinetics that result from the experimental differences described above.

15. Line 280 - How was the oleanolic acid abundance compared between the treatments?

The peaks formed seem too small to affect the abundance of the substrate.

Response: We included the oleanolic acid abundance of the different treatments in **Fig. S17**. We have also redrawn **Fig. 5** and revised the description in the text accordingly. However, it's important to note that, as described in question 14 above, in the leaves expressing NaOSC2+CYP716A419+CYP716E107, no additional oleanolic acid was injected; instead, oleanolic acid was produced through the expression of NaOSC2+CYP716A419. In contrast, the CYP716E107+oleanolic acid combination involved the separate expression of CYP716E107 followed by oleanolic acid injection. These considerations should caution against making direct comparisons in the oleanolic acid content between these two treatments. Additionally, we cannot guarantee identical oleanolic acid uptake by the two leaves, as we cannot control the efficiency of their absorption.

16. Line 285 - Since peak, N10 is not formed with oleanolic acid feeding but is with the CYP716A419, shouldn't N10 be hydroxylated oleanolic aldehyde? Did N10 have the 365 and 189 peaks? Also, 2a-hydroxy-β-amyrin didn't have standards but it was inferred based on fragmentation. Was N10 a triterpene? Was it compared to mass fragmentation libraries?

    Response： N10's EI-MS spectrum exhibits peaks at 189, 203, and 320. Comparing this spectrum with those of oleanolic acid and 6β-hydroxy-oleanolic acid reveals a striking similarity. However, it cannot be a hydroxylated oleanolic aldehyde because N10 displays a strong m/z 320 peak without an m/z 232 peak. By comparing the EI-MS spectra and fragmentation pathways of oleanolic acid, maslinic acid, and betulinic acid, it becomes evident that the presence of a carbonyl group at C28 will yield an m/z 320 peak(**Fig. S10, 11, 15**), whereas an aldehyde group at C28 will yield an m/z 232 peak (**Fig. S9**).The significant presence of oleanolic acid fragmentation features in N10 indicates that it is a derivative of oleanolic acid (peaks at 189, 203, 320). Previously reported in *Centella asiatica*, an enzyme CaCYP716E41, which shares 81% similarity with our NaCYP716E107 protein sequence (**Fig. S16**), when co-expressed CaCYP716E41with GgBAS and CYP716A83 in yeast, yielded two peaks. One of these peaks corresponds to a C6β hydroxylation product of oleanolic acid, similar to our N9, while the other is presumed to be partially derivatized 6β-hydroxy-oleanolic acid (Miettinen et al., 2017). Therefore, we hypothesize that N10 may be a product of a partially derivatized 6β-hydroxy-oleanolic acid.

    The reason why N10 was not detected in tobacco leaves expressing NaCYP716E107 fed with the oleanolic acid substrate is likely due to the substrate entering the cells was too limited, resulting in a low yield of N9.

17. 296 - NaCl is not mimicking salt stress, but is actual salt stress.

    Response： we deleted the “mimicking”

18. Lines 309-310 - GA is involved in growth, but is antagonistic to ABA. the fact that there is a fast response to both, in addition to a response to JA in some cases, which also inhibits growth, may indicate a more complex response, and not just a role in

"growth and development"?

Response： We have rewritten the Results and Discussion sections.

19. Line 322 - 324 - Why were these stresses chosen? Why not heat, or light, or lack of fertilization, or bacteria, or insects?

Response： From the heatmap in **Fig. 1A**, it is evident that, aside from CYP716A419, which is predominantly expressed in flowers, the other genes show higher expression levels in roots than in leaves. Consequently, we opted to focus on stresses associated with roots. Furthermore, our current experimental setup lacks the necessary equipment and maturity to effectively study factors such as heat, light exposure, fertilization deficiency, bacterial infections, or insect infestations targeting roots. We have revised this section and provided the rationale for our stress selection. For further details, please refer to the Results section.

20. Fig. 7 - the statistical analysis method should be chosen. Either a student's t-test, or a Tukey HSD (or any other method). How are we supposed to treat the difference between EOSC1/2 plants in stalk height and diameter? Is it significant or not?

Response： Thanks for suggestion. We deleted the student's t-test result and retained the Tukey HSD result.

21. Lines 383 -385 - The difference between β-amyrin abundances in CYP716A419 plants' theca is not significant. Writing it in the same line with two significant results is misleading. The same is true for lines 385-386 - only one of the three "decreases" in erythrodiol reported is significant, and only two out of three of the oleanolic acid.

Response： We have rewritten the Results and Discussion sections.

22. Fig. 8 - why was the abundance of olivatolic acid not examined? And that of the 2a hydroxy products? Basically, once there are mutants in the native species of the genes I'd expect to see how silencing these genes affects the metabolites in the native system and not only in a heterologous system.

Response： Oleanolic acid is only present in small amounts in flowers, its absence in detection may be attributed to its metabolism into downstream triterpene glycosides **(Yang et al., 2023)**. Although oleanolic acid was not detected, other products of CYP716A419, such as erythrodiol and oleanolic aldehyde, were detected. The absence of 2α-hydroxy products and maslinic acid also could be because they are just the intermediate products. We attempted eliciting roots using ABA, MEJA, or GA3 treatments but were unsuccessful in detecting these compounds. However, we did detect oleanolic acid in roots approximately after induction with MeJA **(Yang et al., 2023)**. Additionally, in roots infected with AMF, we detected low levels of oleanolic acid, maslinic acid, and 6β-hydroxy-oleanolic acid **(Fig. S21)**. This suggests that the inability to detect these compounds may also be due to their low basal levels in *N. attenuata*, and these metabolites may accumulate to detectable levels only during specific growth stages and under particular growth conditions.

23. Line 396 - This is the first time dammarenediol II is mentioned. Why was it searched for?
Response: Dammarenediol II is one of the products of NaOSC1, but its contents in leaves is very low. We supplemented the expression analysis of NaCYP716A419, NaCYP716C87, and NaCYP716E107 genes in different tissues and found that they are mainly expressed in flowers (**Fig. 6A**). Furthermore, the intermediates of these triterpenes are hardly detectable in leaves. Therefore, we reorganized the logic of the Results section and removed the results regarding triterpene contents of leaves.

24. If none of the downstream metabolites were found, how can we assume that the phenotypes seen in the leaves are related to these missing compounds? Especially when the leaf phenotype exists for both the highest and lowest β-amyrin lines?
Response： The increase in β-amyrin levels may potentially cause damage to the waxy structure, but whether its decrease would have an effect is not clear to us.

25. Lines 400 -401: How does a reduction of ~50% in OSC expression reduce a metabolite's abundance by 99%? This is not common even for complete CRISPR knock outs.
Response： As answered in question 23, the content of dammarenediol II in the leaves is extremely low. The reason for the 99% reduction observed here is that although dammarenediol II was detectable in the leaves of the EV plants at a very low concentration (5 ng/g), it was almost undetectable after OSC silencing. Consequently, the transition of dammarenediol II from detectable to undetectable led to the calculation result approaching 99%. Considering the extremely low levels of these triterpene pathway intermediates in the leaves, and supplementary data (**Fig. 6A**) showing that the target genes are predominantly expressed in flowers, we have omitted the leaf content data.

26. Line 417 - CYP716C87 affected only dammarenediol II. CYP716E107 affects compounds in 2/11 conditions. The effects on growth were shown only in competition with other plants. saying that "silencing these biosynthetic genes results in perturbations to in both the triterpene content (Fig. 8) and growth of N. attenuata" is a bit of a stretch.
Response： We have revised the discussion section. Please refer discussion section for details.

27. Lines 505-506 - How is responding to every hormone application tight regulation?
Response： We have revised the discussion section. Please refer discussion section for details.

28. Line 509 - The inference was not tested, and the genes weren't silenced but somewhat reduced.
Response： Please refer discussion section for details.
Line 515 - Was leaf chlorosis quantified? Either by chlorophyl extraction or

spectroscopic methods? If not, how can this be stated as a fact?

Response: We have revised this section.

Lines - 522 - 524 - osc mutants have extremely low β-amyrin compared to control, whereas ctp716a419 mutants have slightly higher β-amyrin. I would expect the EV to have intermediate values between the two. Why are the osc the intermidiates?

Response: We have revised this section. Currently, we cannot provide a definitive answer regarding whether the elevation of β-amyrin decreases pollen activity, the reduction of β-amyrin decreases pollen activity, or if any disruption in β-amyrin content leads to decreased activity. Additionally, β-amyrin serves as a direct precursor to CYP716A419, and they share many downstream steps. This could also be due to the loss of some unknown triterpene of OSC and CYP716A419's common downstream steps. However, the specific roles of downstream triterpenes beyond β-amyrin and oleanolic acid in *Nicotiana attenuata* remain unclear, hence validation is currently not possible. Here, we are merely proposing hypotheses that could be tested in future work.

29. Lines 575 - 578 - if the reason for the phenotypes is autotoxicity, why are competitive conditions necessary to reveal them?

    Response： This is now described in the revised the discussion section.

30. Lines 579 - 580 - this paper does not elucidate the biochemical functions in N. attenuata, but elucidates the biochemical functions of N. attenuata genes in N. benthamiana.

    Response： We have revised the section to distinguish these points.

31. Lines 582 - 583 - The roles of these genes were elucidated in N. benthamiana and not in N. attenuata.

    Response： We have revised this section.

32. Line 584 - nothing was revealed about detoxification by these enzymes. It was suggested along with other equally valid suggestions.

    Response： These points have been tempered in the revised Discussion section.

33. Lines 611 - 612 - Why was the hormonal response profiling performed on the roots? When all the phenotypes and metabolite quantification are performed in the shoot.

    Response： . We have updated the main text to highlight that the hormonal response profiling was conducted on the roots due to predominant expression of these genes in roots. Additionally, it is worth noting that triterpenes accumulate more abundantly in the roots of *N. attenuata* seedlings. During the hormonal treatments, we were uncertain whether silencing these genes would result in a phenotype or where the phenotype would manifest. We also do not rule out the possibility of these metabolites being transferred from the roots to the shoot parts of the plant.

34. Line 631 - why is the 3Ω1 vector cited once for Cárdenas et al., and once for Hong et

al., who cites Cárdenas?

Response： In the main text, we have made the citations to the Cárdenas and Hong references more accurate. 3Ω1 is originally from Cárdenas et al.; Hong et al. utilized a modified version of 3Ω1.

35. Lines 716 - 723 - the statistical analysis section is missing the part of the student's t-tests, which were used in figure 8. Tukey HSD was also not used in Fig. 6A. S8 and S9.

However, most concerningly, no statistics whatsoever seem to have been performed for the first section of the paper. Were they performed and not mentioned?

Response： We have revised the statistical analysis section and included the statistical analysis results from the first part of the study. Please refer to Fig.S3, 5, 6, and Fig. S17 for detailed information.

36. Fig. S10 -This figure seems out of place. Mentioning a result for the first time in the discussion section and heavily relying on a previous publication. Maybe this could be further developed in the results. Or removed altogether?

Response： This figure has now been added to the Results section.

REFERENCE

Carelli, M., Biazzi, E., Panara, F., Tava, A., Scaramelli, L., Porceddu, A., Graham, N., Odoardi, M., Piano, E., Arcioni, S., May, S., Scotti, C., and Calderini, O. (2011). *Medicago truncatula* CYP716A12 is a multifunctional oxidase involved in the biosynthesis of hemolytic saponins. The Plant Cell **23:** 3070-3081.

Fukushima, E.O., Seki, H., Ohyama, K., Ono, E., Umemoto, N., Mizutani, M., Saito, K., and Muranaka, T. (2011). CYP716A subfamily members are multifunctional oxidases in triterpenoid biosynthesis. Plant and Cell Physiology **52:** 2050-2061.

Miettinen, K., Pollier, J., Buyst, D., Arendt, P., Csuk, R., Sommerwerk, S., Moses, T., Mertens, J., Sonawane, P.D., Pauwels, L., Aharoni, A., Martins, J., Nelson, D.R., and Goossens, A. (2017). The ancient CYP716 family is a major contributor to the diversification of eudicot triterpenoid biosynthesis. Nature Communications **8:** 14153.

Misra, R.C., Sharma, S., Sandeep, Garg, A., Chanotiya, C.S., and Ghosh, S. (2017). Two CYP716A subfamily cytochrome P450 monooxygenases of sweet basil play similar but nonredundant roles in ursane- and oleanane-type pentacyclic triterpene biosynthesis. New Phytologist **214:** 706-720.

Moses, T., Pollier, J., Shen, Q., Soetaert, S., Reed, J., Erffelinck, M.L., Van Nieuwerburgh, F.C., Vanden Bossche, R., Osbourn, A., Thevelein, J.M., Deforce, D., Tang, K., and Goossens, A. (2015). OSC2 and CYP716A14v2 catalyze the biosynthesis of triterpenoids for the cuticle of aerial organs of *Artemisia annua*. Plant Cell **27:** 286-301.

Yang, C., Halitschke, R., and O'Connor, S.E. (2023). OXIDOSQUALENE CYCLASE 1 and 2 influence triterpene biosynthesis and defense in Nicotiana attenuata. Plant physiology **194:** 2580-2599.

Yasumoto, S., Seki, H., Shimizu, Y., Fukushima, E.O., and Muranaka, T. (2017). Functional characterization of CYP716 family P450 enzymes in triterpenoid biosynthesis in tomato. Frontiers in Plant Science **8:** 21.

37.

01-Jun-2024

Dr. Ian T Baldwin
Max-Planck-Institut fur chemische Okologie
Department of Molecular Ecology, Hans Knoll strasse 8
Jena D-07745
Germany

RE: Three cytochrome P450 from Nicotiana attenuata play key roles in triterpene biosynthesis

Dear Dr Baldwin

We are pleased to accept your manuscript for publication in the Plant Physiology. This acceptance is contingent on revision based on the comments of our reviewers.

Please highlight all changes and include a detailed annotation to changes to the text, with line numbers, and noting your responses to the comments.

To submit your revised manuscript, click:

Link Not Available

If you cannot return the revised manuscript within 8 weeks of receipt, please let us know. Otherwise, we will assume that you have elected not to revise the manuscript and are withdrawing it.

Thank you for allowing us to review your work. We look forward to hearing from you soon.

Sincerely, Editor

Plant Physiology

-- Reviewer comments:

Reviewer #1 (Comments for the Author):

I appreciate the revisions done by the authors, which have certainly improved the presentation and overall quality of the manuscript. There are still some remaining problems to be addressed as specified below:

1. The title is still a bit vague and requires further modification to be more specific and reflect the actual content of the article. The one-sentence summary might be more appropriate for the title.
2. The second paragraph regarding triterpene biosynthesis and scaffold formation in the Introduction section needs to be rewritten. It seems that there are only a few triterpene scaffolds known so far based on the current presentation, which is certainly not the case. It makes more sense for the authors to focus on pentacyclic triterpene scaffolds when writing introduction.
3. When depicting the structures of those putatively identified compounds (e.g. N3, N8, A2, A7) in the Figures, a pair of bracket needs to be included to indicate the "putative" nature of the structures included.
4. Fig. 1A and fig. S1, "Heatmap" is not a suitable description for the color key and needs changing, e.g. use "relative expression"
5. Fig.5B, structure displayed is incomplete.

Reviewer #2 (Comments for the Author):

The authors significantly altered the manuscript in line with my comments. I especially appreciate the authors presenting the data breakdown in Figure S3 and similar figures. The results are now presented with less over-interpretation. I still think that trying to understand the function of a gene by reducing its expression by 50% (and not examining how much of the metabolite is actually in the leaf) is not the right approach when CRISPR is so available (a comment which was not answered in mt previous review), however, now that the authors do not make cutting conclusions based on it, but suggest broader possibilities, I think the second part of the N. attenuata experiments presents results which the readers may interpret as they wish.
I do think that following the many additions to the text made in this version, the text flows a bit less. Lines 248-266 are a good

example of this, where detailing all the fragments is a bit too much maybe, and the distinctive fragment, which is the one used for characterization should be retained while the rest are removed.

Specific comments:

Throughout the entire manuscript there is wrong usage of italics and capitals in gene and protein names. Whenever talking about expression of genes, even when this is in benthamiana, the gene name should be italicized. Only when talking about the actual protein should it be capitalized without italics.
I think that the authors should use scientific convention for gene and protein names. Using lowercase letters to "distinguish" the gene from the enzyme, as stated in reply 3, does not help, since readers might assume those are mutants.

In places where peaks were extracted, the figure legend should mention what ion was used for quantification. When the chromatograms are shown the legend (or figure itself) should indicate if this is an extracted ion chromatogram or TIC, and what is the extracted ion.

Figure S3 - In all the samples where there isn't a distinct peak, "nd" is brought as the value. However, in Fig. S4, we can see that there is a peak in the EV+DMSO sample in the same RT as the N2 compound. This is obviously a very small peak, but the difference between 0 and any number is infinite. I would try to extract the peak size when it exists, even if it is very small, so a proper statistical analysis can be performed.

Figure S4 -what is the authentic standards for? It seems in panel B to have peeks for both -amyrin and erythrodiol, and in panel A there are combinations of both -amyrin and lupeol and erythrodiol and oleanolic acid(?).
what mass was extracted to see the peaks? Assuming this is not TIC?

Figure 2A - please indicate which standard was used. There are several peaks, and it is not clear which standard was used (the same goes for 2B and Fig3)

Fig. 1-3 - in Fig. 1 and 2, expressing CYP716A419 with OSC2 leads to production of oleanolic acid, aldehyde and erythrodiol. However, in Fig. 3B, this same combination leads only to oleanolic acid. What is the difference between the assays that led to this difference?
Also, in Fig. S3 this combination is shown to always lead to formation of the 3 compounds.

Fig S17 - should "Turkey's test" be Tukey?

Lines 304 - 305 - the statistical test comparing to 0 is not able to determine if the peak is different than control. There are ways to compare this - a non-parametric test or even a chi square where presence/ absence is scored. When we see a peak forming where there was not one before, it is tempting to think we do not need statistics - it's black and white, it's either here or not. That is where a chi square test will tell us is we have enough samples to come to this conclusion, taking into account the number of comparisons we've made. I don't think it is correct to talk about changes if this is not statistically supported.

Lines 324 - 326 - what does it mean that extracts were supplemented in vivo? From the figure it looks like the genes were expressed together.

Figure 8 - There are very striking differences in the leaf morphology shown in Fig, 8A. were the metabolites quantified in the leaves? Expression can be important of course, but when talking about the effects on the growth phenotype, I'd expect to know how -amyrin and the products of CYP716A419 were altered in the strong phenotype lines.

β-Amyrin is a major cuticular wax component in many plant species, including tomato. The phenotypes of the CYP716A419 are very similar to cuticle mutants - cutin mutants have elongated and deformed leaves, and both cutin and cuticular wax mutants have strong effects on pollen viability. Could the β-Amyrin accumulation be affecting cuticular properties?

Methods - what about the N. benthamiana growth conditions?

Line 665 - Is the concentration of 0.4 for the gene combination 0.4 of each, or is the final OD of the buffer 0.4?

Lines 713 - 714 - The samples were not ground? Is this assuming that all these metabolites are excreted on the surface of the tissue? Why were the pollen samples ground then?

Statistical analysis - As I wrote previously in this review, I don't think a group of samples which are all not detected can be compared using an ANOVA or Student's t-tests or Tukey's HSD. In any case, this section should say what was done in cases with such samples. Were they compared in a Tukey's test? why do thy not have letters on the graphs then? Were they not included in the comparison? Then why are there letters, indicating a statistical test in the graphs (12 of the panels in Fig. S3 for example have values in only one samples, and the letter "a" below it).

Manuscript ID: PP2024-RA-00711D

Title: *Three cytochrome P450 from Nicotiana attenuata play key roles in triterpene biosynthesis*

Dear Editors and Reviewers,

Thank you for your letter and the reviewers' comments on our manuscript. We sincerely appreciate the valuable feedback provided by the reviewers regarding our manuscript with the ID: PP2024-RA-00711D. The quality of our manuscript has been significantly enhanced by incorporating the insightful comments we received. We are deeply grateful for the intellectual generosity of the editors and reviewers.
The point-by-point response to the reviewers' comments is as follows:

Reviewer #1 (Comments for the Author):

I appreciate the revisions done by the authors, which have certainly improved the presentation and overall quality of the manuscript. There are still some remaining problems to be addressed as specified below:

1. **The title is still a bit vague and requires further modification to be more specific and reflect the actual content of the article. The one-sentence summary might be more appropriate for the title.**

   Response: we changed the title to "Roles of Three CYP716 Enzymes in Triterpenes Biosynthesis and Their Potential Impact on Growth and Development"

2. **The second paragraph regarding triterpene biosynthesis and scaffold formation in the Introduction section needs to be rewritten. It seems that there are only a few triterpene scaffolds known so far based on the current presentation, which is certainly not the case. It makes more sense for the authors to focus on pentacyclic triterpene scaffolds when writing the introduction**
   Response: In the Introduction section, we have rewritten the second paragraph regarding triterpene biosynthesis and scaffold formation. Please refer to lines 56~62 for details.

3. **When depicting the structures of those putatively identified compounds (e.g. N3, N8, A2, A7) in the Figures, a pair of bracket needs to be included to indicate the "putative" nature of the structures included.**
   Response: many thanks for the suggestions, we add "putative" to these compounds.

4. **Fig. 1A and fig. S1, "Heatmap" is not a suitable description for the color key and needs changing, e.g. use "relative expression"**
   Response: we changed "heatmap" to "relative gene expression level".

5. **Fig.5B, structure displayed is incomplete.**
Response: many thanks for the careful reading, we have addressed this mistake.

Reviewer #2 (Comments for the Author):

1. **The authors significantly altered the manuscript in line with my comments. I especially appreciate the authors presenting the data breakdown in Figure S3 and similar figures. The results are now presented with less over-interpretation. I still think that trying to understand the function of a gene by reducing its expression by 50% (and not examining how much of the metabolite is actually in the leaf) is not the right approach when CRISPR is so available (a comment which was not answered in mt previous review), however, now that the authors do not make cutting conclusions based on it, but suggest broader possibilities, I think the second part of the N. attenuata experiments presents results which the readers may interpret as they wish.**
Response: many thanks for the comments. The primary reason for not choosing CRISPR for functional validation is that we currently lack the necessary conditions for its implementation. Although CRISPR has numerous advantages in gene editing, and we hope to use it to further knock out these target genes to explore the underlying mechanisms, its implementation requires mature transformation tools, transformation methods, long-term screening, and a strong molecular biology background. These requirements are not fully met in our current setup. In comparison, while VIGS is less stable and effective than CRISPR, it offers a significant advantage in terms of time. VIGS can quickly determine whether a gene is worth further investigation. In a laboratory where conditions and technical costs are limited, VIGS can still play a valuable role. In fact, before the advent of CRISPR technology, VIGS had been used for a considerable time in gene function studies. Despite its imperfections, it indeed partly serves its purpose. Regarding this study, although VIGS cannot fully clarify the function and mechanism of the gene, the results are sufficient to indicate that NaCYP716A419 affects the growth of tobacco (*N. attenuata*). Silencing NaCYP716A419 impacts the biosynthesis of triterpenoids, suggesting that this gene is worth further investigation. Once we establish more stable gene knockout tools, we can further explore its functions and mechanisms.

2. **I do think that following the many additions to the text made in this version, the text flows a bit less. Lines 248-266 are a good example of this, where detailing all the fragments is a bit too much maybe, and the distinctive fragment, which is the one used for characterization should be retained while the rest are removed.**
Response: We removed the superfluous descriptions.

Specific comments:

1. **Throughout the entire manuscript, there is wrong usage of italics and capitals in gene and protein names. Whenever talking about expression of genes, even when this is in benthamiana, the gene name should be italicized. Only when talking about the actual protein should it be capitalized without italics. I think that the authors should use scientific**

conventions for gene and protein names. Using lowercase letters to "distinguish" the gene from the enzyme, as stated in reply 3, does not help, since readers might assume those are mutants.

Response: We modified the names of proteins and genes in italics.

2. **In places where peaks were extracted, the figure legend should mention what ion was used for quantification. When the chromatograms are shown the legend (or figure itself) should indicate if this is an extracted ion chromatogram or TIC, and what is the extracted ion.**

   Response: We added information about extracting ions to the figure legends.

3. **Figure S3 - In all the samples where there isn't a distinct peak, "nd" is brought as the value. However, in Fig. S4, we can see that there is a peak in the EV+DMSO sample in the same RT as the N2 compound. This is obviously a very small peak, but the difference between 0 and any number is infinite. I would try to extract the peak size when it exists, even if it is very small, so a proper statistical analysis can be performed.**

   Response: The result in Fig. S4 is the extracted particle flow diagram of SIM (189,203 and 216 extract the peaks here). The peak is very small, with an intensity of only 1 K count, which is 200 times lower than the peak of EV+erythrodiol. This peak also has no obvious m/z 216, so the quantitative integration of m/z (203,216) can not be used to obtain the peak area.

4. **Figure S4 -what is the authentic standards for? It seems in panel B has peeks for both □-amyrin and erythrodiol, and in panel A there are combinations of both □-amyrin and lupeol and erythrodiol and oleanolic acid(?). what mass was extracted to see the peaks? Assuming this is not TIC?**

   Response: We have added information about the extracted ions and the authentic standards used in the figure legend.

5. **Figure 2A - please indicate which standard was used. There are several peaks, and it is not clear which standard was used (the same goes for 2B and Fig3)**

   Response: In the figure legend, we have included information about the authentic standards.

6. **Fig. 1-3 - in Fig. 1 and 2, expressing CYP716A419 with OSC2 leads to production of oleanolic acid, aldehyde and erythrodiol. However, in Fig. 3B, this same combination leads only to oleanolic acid. What is the difference between the assays that led to this difference? Also, in Fig. S3 this combination is shown to always lead to the formation of the 3 compounds.**

   Response: As shown in Figures 1 and 2, CYP716A419 with OSC2 leads to the production of oleanolic acid, aldehyde, and erythrodiol. However, the oleanolic acid and aldehyde could not be adequately separated, which would affect the identification of subsequent oxidation products. Therefore, when adding other CYP450 enzymes based on NaOSC2+CYP716A419, we divided the extraction process into two parts as described in the methods section.

   When extracting samples, we first saponified the samples and then used hexane to extract the alkaline layer. Subsequently, we acidified the saponified solution with hydrochloric acid and used hexane to extract the acidic layer. In the samples shown in Figures 1 and 2 and the

supplementary Figure S3, the alkaline and acidic layers were combined for detection. However, in Figure 3b, we analyzed the alkaline and acidic layers separately.

Figure 3b focuses on whether CYP716C87 can oxidize oleanolic acid to maslinic acid. Therefore, we only presented the detection results of the acidic layer in this figure. We have added an explanation in the figure legend of Figure 3b to indicate that the results shown are only for the acidic layer.

7. **Fig S17 - should "Turkey's test" be Tukey?**
   Response: Thanks for this correction.

8. **Lines 304 - 305 - the statistical test comparing to 0 is not able to determine if the peak is different than control. There are ways to compare this - a non-parametric test or even a chi square where presence/ absence is scored. When we see a peak forming where there was not one before, it is tempting to think we do not need statistics - it's black and white, it's either here or not. That is where a chi-square test will tell us if we have enough samples to come to this conclusion, taking into account the number of comparisons we've made. I don't think it is correct to talk about changes if this is not statistically supported.**
   Response: Thank you for this valuable suggestion. However, typically, Chi-square analysis requires a large sample size, and one important assumption is that the expected count in each cell should be at least 5. For these analyses, each treatment group has only 3 replicates, with a few having 5. The small sample size results in a very low expected count, sometimes almost zero for Chi-square analysis. Here, the analysis result for N2 serves as an example. The original data are shown in **Table 1**. We can see the expected count of most cells is less than 5 （**Table 2** and **Table 3**）. Although the Chi-square test shows significance, some researchers suggest that Fisher's Exact Test is more suitable for small expected count data. However, since most of our data only has 3 replications, based on your suggestions we conducted anon-parametric analysis instead. We chose to assign a peak area of zero to combinations where no peak was detected and then performed the Kruskal-Wallis test. The Kruskal-Wallis test is a non-parametric statistical test used to compare two or more independent samples to determine if they come from the same distribution. It is the non-parametric counterpart of the one-way analysis of variance (ANOVA), used when the data do not meet the normality or homogeneity of variance assumptions required for ANOVA. The results indicate that these findings are significant. Please see Fig. S4, S5, S6 and S17 for details.

Table 1 Original dataset of N2.

expression combinations	groups	peak area	NO/YES
Empty vector	A	0	no
Empty vector	A	0	no
Empty vector	A	0	no
Empty vector	A	0	no
Empty vector	A	0	no
NaOSC2	B	0	no
NaOSC2	B	0	no
NaOSC2	B	0	no

NaOSC2	B	0	no
NaOSC2	B	0	no
NaOSC2+NaCYP88B4	C	0	no
NaOSC2+NaCYP88B4	C	0	no
NaOSC2+NaCYP88B4	C	0	no
NaOSC2+NaCYP88B4	C	0	no
NaOSC2+NaCYP88B4	C	0	no
NaOSC2+NaCYP716D93	D	0	no
NaOSC2+NaCYP716D93	D	0	no
NaOSC2+NaCYP716D93	D	0	no
NaOSC2+NaCYP716D93	D	0	no
NaOSC2+NaCYP716D93	D	0	no
NaOSC2+NaCYP716A420	E	0	no
NaOSC2+NaCYP716A420	E	0	no
NaOSC2+NaCYP716A420	E	0	no
NaOSC2+NaCYP716A420	E	0	no
NaOSC2+NaCYP716A420	E	0	no
NaOSC2+CYP716D94	F	0	no
NaOSC2+CYP716D94	F	0	no
NaOSC2+CYP716D94	F	0	no
NaOSC2+CYP716D94	F	0	no
NaOSC2+CYP716D94	F	0	no
NaOSC2+NaCYP716E108	G	0	no
NaOSC2+NaCYP716E108	G	0	no
NaOSC2+NaCYP716E108	G	0	no
NaOSC2+NaCYP716E108	G	0	no
NaOSC2+NaCYP716E108	G	0	no
NaOSC2+NaCYP716H6	H	0	no
NaOSC2+NaCYP716H6	H	0	no
NaOSC2+NaCYP716H6	H	0	no
NaOSC2+NaCYP716H6	H	0	no
NaOSC2+NaCYP716H6	H	0	no
NaOSC2+NaCYP716A419	I	237537	yes
NaOSC2+NaCYP716A419	I	363600	yes
NaOSC2+NaCYP716A419	I	401484	yes
NaOSC2+NaCYP716A419	I	377333	yes
NaOSC2+NaCYP716A419	I	238835	yes
NaOSC2+NaCYP716C87	G	0	no
NaOSC2+NaCYP716C87	G	0	no
NaOSC2+NaCYP716C87	G	0	no
NaOSC2+NaCYP716C87	G	0	no
NaOSC2+NaCYP716C87	G	0	no
NaOSC2+NaCYP716E107	K	0	no

NaOSC2+NaCYP716E107	K	0	no
NaOSC2+NaCYP716E107	K	0	no
NaOSC2+NaCYP716E107	K	0	no
NaOSC2+NaCYP716E107	K	0	no

Table 2 **Expected Count of the cell.**

groups * no/yes Crosstabulation

			no/yes		Total
			no	yes	
**groups**	A	Count	5	0	5
		Expected Count	4.5	0.5	5
	B	Count	5	0	5
		Expected Count	4.5	0.5	5
	C	Count	5	0	5
		Expected Count	4.5	0.5	5
	D	Count	5	0	5
		Expected Count	4.5	0.5	5
	E	Count	5	0	5
		Expected Count	4.5	0.5	5
	F	Count	5	0	5
		Expected Count	4.5	0.5	5
	G	Count	10	0	10
		Expected Count	9.1	0.9	10
	H	Count	5	0	5
		Expected Count	4.5	0.5	5
	I	Count	0	5	5
		Expected Count	4.5	0.5	5
	K	Count	5	0	5
		Expected Count	4.5	0.5	5
Total		Count	50	5	55
		Expected Count	50	5	55

Table 3 **Chi-Square Test result**

Chi-Square Tests

	Value	df	Asymptotic Significance (2-sided)	Exact Sig. (2-sided)
Pearson Chi-Square	55.000[a]	9	.000	.000
Likelihood Ratio	33.510	9	.000	.000
Fisher's Exact Test	23.242			.000
N of Valid Cases	55			

a. 19 cells (95.0%) have expected count less than 5. The minimum expected count is .45.

9. **Lines 324 - 326 - what does it mean that extracts were supplemented in vivo? From the figure it looks like the genes were expressed together.**
   Response: We corrected the description of the sentences.

10. **Figure 8 - There are very striking differences in the leaf morphology shown in Fig, 8A. were the metabolites quantified in the leaves? Expression can be important of course, but when talking about the effects on the growth phenotype, I'd expect to know how □-amyrin and the products of CYP716A419 were altered in the strong phenotype lines. β-Amyrin is a major cuticular wax component in many plant species, including tomato. The phenotypes of the CYP716A419 are very similar to cuticle mutants - cutin mutants have elongated and deformed leaves, and both cutin and cuticular wax mutants have strong effects on pollen viability. Could the β-Amyrin accumulation be affecting cuticular properties?**

    Response: Many thanks for these thoughtful comments. In the leaves where CYP716A419 was silenced, there was indeed a significant increase in β-Amyrin compared to the EV group, as evidenced by the data in the new Fig. 8. The accumulation of β-amyrin resulting from the silencing of CYP716A419 may have contributed to the disruption of the cuticular wax structure, which we initially hypothesized as a factor driving the observed phenotypic changes. However, despite the significant increase in β-amyrin observed in the leaves of CYP716A419-VIGS plants compared to EV, the difference was not substantial compared to CYP716C87-VIGS and CYP716E107-VIGS.

    This discrepancy is perplexing because if the disruption of the cuticular wax structure caused by β-amyrin is the primary reason, then why don't CYP716C87-VIGS and CYP716E107-VIGS show significant leaf phenotype changes? We speculate that other unknown triterpenoids may be involved in the regulation of cuticular wax synthesis, as the substrates of CYP716A419 are not limited to β-amyrin alone. Lupeol and lupanediol are also substrates, but their content in *N. attenuata* tissues is relatively low and typically increases only after herbivore induction, making them challenging to detect. Additionally, it remains uncertain whether CYP716A419 can catalyze additional substrates, such as other undisclosed triterpenoids or steps related to wax synthesis and even steps related to gibberellin synthesis. It's also plausible that it's not β-amyrin itself but downstream, more complex triterpenoids that are influencing the observed effects. These avenues will serve as directions for further research and discussion.

11. **Methods - what about the N. benthamiana growth conditions?**
 Response: We added the growth conditions of *N. benthamiana* to the method.

12. **Line 665 - Is the concentration of 0.4 for the gene combination 0.4 of each, or is the final OD of the buffer 0.4?**
 Response: We have revised the description involved here. The concentration of 0.4 for the gene combination typically refers to the concentration of the individual components within the gene combination, rather than the final optical density (OD) of the buffer.

13. **Lines 713 - 714 - The samples were not ground? Is this assuming that all these metabolites are excreted on the surface of the tissue? Why were the pollen samples ground then?**
 Response: Simple triterpenes are present in the epidermal wax of plants, especially in the inner layer of wax. Grinding and extraction should facilitate a more thorough extraction of these compounds. In this study, leaves and flowers of *N. attenuata* were not subjected to grinding extraction. This decision was made because *N. attenuata* tissues are rich in metabolites, but triterpenes are present in relatively low concentrations within this diverse metabolic pool. Additionally, *N. attenuata* is covered with abundant secretory trichomes throughout its body, and grinding would result in the rupture of these trichomes, releasing a large number of metabolites. Furthermore, grinding would also result in the extraction of high levels of chlorophyll and sterols, among other metabolites far more abundant than the triterpenes, effectively masking their presence during detection. Extraction of ground leaves did not result in the detection of any simple triterpene compounds in *N. attenuata*, so we used the hexane wash. However, pollen metabolites have a relatively simple metabolic background and lack trichomes. Therefore, grinding extraction would not introduce significant amounts of interfering metabolites, allowing for the detection of triterpenes. To ensure the detection of these enzyme products as much as possible, extraction of ground pollen and theca was used for these analyses.

14. **Statistical analysis - As I wrote previously in this review, I don't think a group of samples that are all not detected can be compared using an ANOVA or Student's t-tests or Tukey's HSD. In any case, this section should say what was done in cases with such samples. Were they compared in a Tukey's test? why do thy not have letters on the graphs then? Were they not included in the comparison? Then why are there letters, indicating a statistical test in the graphs (12 of the panels in Fig. S3 for example have values in only one samples, and the letter "a" below it).**
 Response: Thank you for pointing out this issue, which is crucial for modifying our statistical analysis. In fact, during our statistical analysis, we treated the samples where metabolites were not detected as having peak areas of zero and compared them with samples where metabolites were detected. Then, we replaced the statistical labels of the samples where metabolites were not detected with "nd" (not detected). We have re-conducted the statistical analysis on the samples and included the Kruskal-Wallis test.

06-Jul-2024
Dr. Ian T Baldwin
Max-Planck-Institut fur chemische Okologie
Department of Molecular Ecology, Hans Knoll strasse 8
Jena D-07745
Germany

MSID: PP2024-RA-00711DR1
MS TITLE: Roles of Three CYP716 Enzymes in Triterpenes Biosynthesis and Their Potential Impact on Growth and Development

Dear Dr. Ian Baldwin:

I am pleased to inform you that your revised manuscript "Roles of Three CYP716 Enzymes in Triterpenes Biosynthesis and Their Potential Impact on Growth and Development" has been accepted for publication in Plant Physiology. Your manuscript will be checked for consistency with journal formatting and image standards by one of our Science Editors, who will contact you shortly. They may have a number of queries and suggestions for you. Please keep in mind that they work to increase the online visibility of your article and its attraction for readers from the widest of backgrounds. You will have the opportunity to review any revisions, and I hope you will work constructively with them to get the most out of your research. You will also have the opportunity to review all changes when you receive the author proofs for your article.

Once validated for publication, your article will be sent to a Science Editor for a final review of your manuscript and source files. After your final files are uploaded, your article will appear in the next available issue and the online Preview version of your article will be posted after your signed Copyright and Checklist forms and final source files are received.

Please be sure to include the following in your manuscript file:
Harvard style citations
List of all author contributions and funding information at the beginning of manuscript after the titles and author list.
List all supplemental materials associated with the article, with titles, at the end of the ms, before the references and acknowledgments section.
Complete Figure Legends for all figures appearing in the article listed before the Literature Cited.

Thank you and congratulations on your Plant Physiology paper!

Sincerely,
Editor
Plant Physiology

=====
FIRST AUTHOR PROFILES
=====

We recognize the hard work that goes into being First Author of a Plant Physiology paper by publishing a "First Author Feature" for each First Author alongside your paper.

If the first author(s) would like to submit information for a feature on Facebook and Twitter, fill out the form by clicking on the link below:

Plant Physiology First Author Feature Form: https://forms.office.com/r/ZBCGZfhm8F
If you cannot access the form through the link above, please contact Rachel at rbelsky@aspb.org.

To upload an image of yourself to accompany your profile, please put your photo in the correct journal folder at the link below. You must label your image: Author First Name Last Name Article Title.

First Author Profile Image Dropbox: https://www.dropbox.com/scl/fo/xz029p7qjr2jml2rp2kmq/h?dl=0&rlkey=6fkxcaqmxx35ap152b2osy1fz

=====
IMPORTANT REMINDER: PEER REVIEW REPORTS
=====

If you opted to publish a peer review report along with your article during the original submission process, it will be prepared by the editorial staff and publicly posted with your manuscript, inside the zip file that contains any other supplemental material. As a reminder, the peer review report is a public record of all comments from editors and reviewers, as well as your prior responses, as you received them in the decision letters for each draft of your manuscript. If you agreed to publish this report and have changed your mind, or are not sure if you selected this option, please contact the editorial office as soon as possible before signing the license agreement from our publisher.

-- Reviewer comments:
